# Axioms of Diagnostic Urgency: A Characterization Theorem

**DOI:** 10.3390/e28080853

**Published:** 2026-07-31

**Authors:** Pedram Safari, Amal Mohamed, Shuhan He

**Affiliations:** 1 MGH Institute of Health Professions, Boston, MA 02129, USA; psafari@mghihp.edu; 2 Department of Emergency Medicine, Massachusetts General Hospital, Harvard Medical School, Boston, MA 02114, USA; abmohamed@mgh.harvard.edu

**Keywords:** diagnostic urgency, entropy, information theory, multiattribute utility, emergency medicine, representation theorem, clinical decision support

## Abstract

Diagnostic prioritization, deciding which diagnoses in a differential should be evaluated first, is fundamental to emergency medicine but lacks a formal foundation. We model diagnostic urgency using four parts of a diagnostic scenario: outcome distribution, irreversibility, preventable treatment benefit, and time lost as treatment effectiveness decays. We propose six axioms: four monotonicity conditions, coordinate independence, and continuity. We then prove a representation theorem: any urgency function satisfying these axioms and the stated regularity conditions can be represented by an additive score with one component for each part. We also give one axiom-valid construction using expected severity, an irreversibility index, directional preventable expected severity, and hyperbolic temporal decay. The theorem-aligned score is the primary urgency measure throughout; we report a severity-weighted entropy score separately as an exploratory enrichment, characterizing exactly the simplex sub-region on which it respects the severity-dominance axiom. The theorem-aligned score correlates with DALY-derived condition weights across 42 emergency conditions (r=0.934) and, in a retrospective MIMIC-IV v2.2 condition-level criterion-validity analysis, with in-hospital mortality (r=0.684, 95% CI [0.494, 0.837]) and ICU admission (r=0.637); the entropy enrichment correlates with mortality at r=0.747, but its incremental value over the theorem-aligned score is not statistically significant ($\Delta R^{2}=0.04$, p=0.11). The score separates higher- from lower-acuity conditions (AUC = 0.96 across 42 conditions). The framework gives a formal basis for studying the clinical principle of “ruling out the dangerous diagnoses first”; it is not yet a prospectively validated clinical decision rule.

## 1. Introduction

In emergency medicine, physicians routinely prioritize some diagnoses within a differential for rapid evaluation. A patient presenting with acute headache may have subarachnoid hemorrhage (case fatality ~35%) or tension-type headache (benign, self-limited). Before the diagnosis is confirmed, the physician must decide which possibilities to investigate first. Every medical trainee learns this idea, and clinicians recognize it as essential to patient safety, but the basis for the ordering has not been formally characterized: what makes one diagnosis more urgent to evaluate than another?

Existing mathematical frameworks address adjacent problems. Information-theoretic approaches formalize diagnostic uncertainty: Bayesian test selection minimizes expected posterior entropy [1,2], and information theory has been applied directly to diagnostic testing to quantify the uncertainty that a test result removes [3]. Recent clinical entropy-removal work has applied Shannon entropy to diagnostic decision tools and individual clinical features, showing how diagnostic findings and algorithms reduce uncertainty beyond conventional accuracy measures [4,5]; a recent decision-theoretic review surveys this literature and notes that entropy has not yet been translated into an operational, evidence-based decision-support framework [6]. The Global Burden of Disease framework quantifies health loss at the population level via disability-adjusted life years [7]. Clinical acuity tools operate at the patient level: APACHE II and SOFA quantify physiologic severity in ICU populations [8,9]; the Emergency Severity Index sorts patients by triage acuity [10]. Across 33 triage systems, established tools show moderate-to-good but highly variable validity [11], and machine-learning triage models are now active in emergency departments [12]. The National Early Warning Score detects deterioration [13], and clinical decision rules (Wells criteria, HEART score) estimate the probability of specific diagnoses [14,15]. Multi-criteria decision analysis (MCDA) provides structured methods for evaluating healthcare interventions across multiple attributes [16,17], is now embedded in regulatory benefit-risk decisions [18], and predominantly uses linear additive aggregation without justifying its axiomatic basis [19]; it addresses resource allocation across interventions rather than within-differential diagnostic prioritization. None of these frameworks addresses the diagnosis-level question: given that a differential diagnosis exists, how urgent is it to confirm or exclude each candidate?

We formalize diagnostic urgency as a real-valued function on a four-factor product space of diagnostic scenarios and characterize its structure with axioms. The approach follows classical representation theorems in decision theory [20,21,22]: we state clinically motivated requirements and derive their mathematical consequences. The main result (Theorem 1) shows that any function satisfying the axiom set and the stated regularity conditions decomposes additively across four dimensions. The four attributes, outcome trajectory, irreversibility, treatability, and time-sensitivity, are identified by domain analysis of diagnostic scenarios (Section 2.2). The key result is the additive aggregation rule, which follows from coordinate independence and continuity on the connected product domain once essential coordinates and regularity conditions are in place. We exhibit one axiom-valid construction, anchor the empirical analyses on the theorem-aligned score, compare it with DALY-derived condition weights and with MIMIC-IV outcomes, report a severity-weighted entropy score separately as exploratory enrichment, and discuss implications for clinical reasoning.

## 2. Preliminaries

### 2.1. Outcome States and Severity

Let $\Omega =\{\omega_{1},\omega_{2},\ldots ,\omega_{K}\}$ be a finite set of health outcome states, ordered by severity:

$$\omega_{1}{≻}_{s}\omega_{2}{≻}_{s}\ldots {≻}_{s}\omega_{K}$$

where ${≻}_{s}$ denotes “strictly more severe than.” We fix K=5 with the canonical health states:

Ω={death,severedisability,moderatedisability,milddisability,fullrecovery}

A severity function s:Ω→[0,1] assigns weights consistent with the ordering: $s\left(\omega_{1}\right)=1.00>s\left(\omega_{2}\right)=0.55>s\left(\omega_{3}\right)=0.35>s\left(\omega_{4}\right)=0.10>s\left(\omega_{5}\right)=0.00$. These weights are derived from the Global Burden of Disease 2019 disability weight scales [7] by averaging across GBD health states within each severity tier (e.g., $s_{2}=0.55$ averages GBD disability weights for severe sequelae including major stroke, paraplegia, and severe TBI; $s_{4}=0.10$ averages weights for mild sequelae including mild anxiety and minor chronic pain).

**Remark** **1.** *The five-state partition is a modeling choice. The results generalize to any finite K≥2 with strictly ordered severity weights. The essential requirement is a finite, totally ordered outcome space equipped with a severity function. Because the theorem-aligned trajectory component is an expectation $\sum_{k}p_{k}s\left(\omega_{k}\right)$, it is invariant under any mean-preserving refinement of the severity partition; consequently neither the four-factor product structure of D nor the condition ranking depends materially on the granularity of *Ω*. We verify this robustness empirically in Section 6.4.*

### 2.2. The Space of Diagnostic Scenarios

A diagnostic scenario specifies the complete clinical profile of a single diagnosis within a differential. It is a tuple d=(P,C,E,h) drawn from a product space D with four factors:

**Factor 1** (Natural history distribution)**.** *$P\in \Delta^{\circ }\left(\Omega \right)$, the interior of the (K−1)-dimensional probability simplex. Here $\Delta \left(\Omega \right)=\left\{P=\left(p_{1},\ldots ,p_{K}\right):p_{k}\geq 0,\;\sum_{k=1}^{K}p_{k}=1\right\}\subset R^{K}$, and $\Delta^{\circ }\left(\Omega \right)=\left\{P\in \Delta \left(\Omega \right):p_{k}>0\;\mathrm{for}\;\mathrm{all}\;k\right\}$. This excludes boundary distributions, including degenerate distributions that assign probability 1 to a single outcome. No real diagnosis has a literally certain outcome; even conditions colloquially described as “uniformly fatal” have nonzero survival rates in clinical data. P describes the distribution of health outcomes if the diagnosis is present but unidentified (the “missed diagnosis” distribution). $\Delta^{\circ }\left(\Omega \right)$ is a convex open subset of $R^{K}$, hence connected.*

**Factor 2** (Irreversibility structure)**.** *$C\in C={\left[0,1\right]}^{2}$, parameterized by the pair $\left(c,w_{c}\right)$ where c∈[0,1] is the probability of transitioning to a permanent health state, and $w_{c}\in \left[0,1\right]$ is the disability weight of that permanent state. C is compact and connected under the standard topology on ${\left[0,1\right]}^{2}$.*

**Factor 3** (Treatment effect)**.** *E∈E=Δ(Ω)×Δ(Ω), the pair $\left(P^{\mathrm{tx}},P^{\mathrm{untx}}\right)$ of outcome distributions under timely treatment and without treatment, respectively. The treatment effect is the directional reduction in expected severity produced by timely treatment. E carries the product topology from two copies of Δ(Ω) and is compact and connected.*

**Remark** **2.** *The natural history distribution P (Factor 1) describes outcomes when the diagnosis is entirely missed: the patient receives no targeted intervention. The untreated distribution $P^{\mathrm{untx}}$ in Factor 3 can also describe outcomes when the diagnosis is known but treatment is withheld or unavailable. These are conceptually distinct: a missed diagnosis of pulmonary embolism results in no anticoagulation and possible delayed recognition, while a known but untreated PE (e.g., due to contraindications) involves monitoring and supportive care. In practice, P and $P^{\mathrm{untx}}$ are correlated but not identical.*

**Factor 4** (Temporal half-life)**.** *h∈T=[0,∞] is the half-life of treatment effectiveness. We equip [0,∞] with the order topology, under which it is compact and connected (homeomorphic to [0,1] via h↦1/(1+h)).*

The full diagnostic scenario space is the product:

$$D=\Delta^{\circ }\left(\Omega \right)\times C\times E\times T$$

Each factor space is equipped with its standard topology. The combined space D carries the product topology. Each factor is connected; therefore D is connected (Munkres [23], Theorem 23.6).

**Figure 1:**
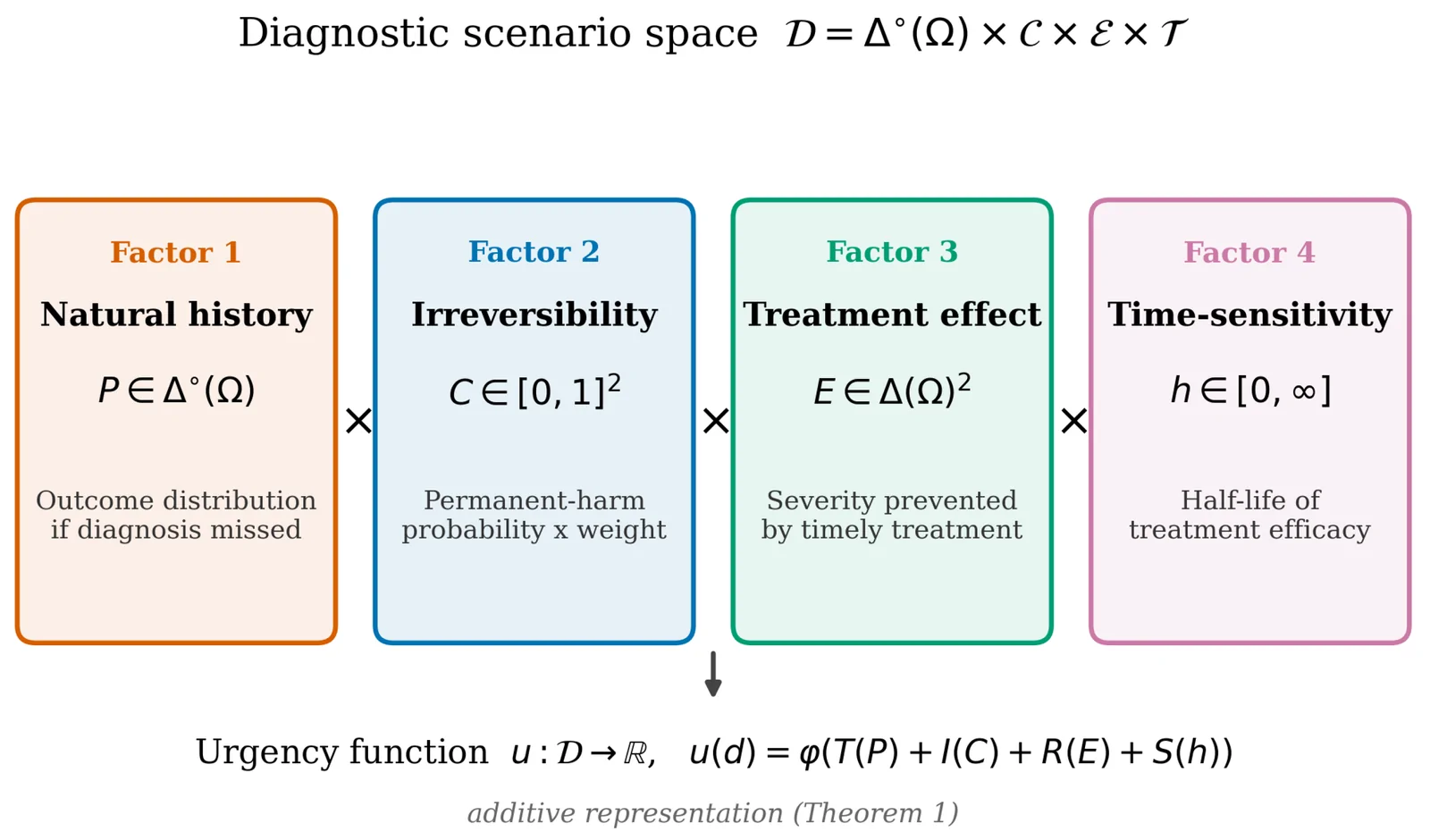
Schematic of the four-factor diagnostic scenario space $D=\Delta^{\circ }\left(\Omega \right)\times C\times E\times T$, with clinical examples for each factor. The urgency function *u* decomposes additively across the four factors (Theorem 1).

**Remark** **3.** *The four factors of D correspond to four sources of variation in a diagnostic scenario: (i) what happens if the diagnosis is missed, (ii) which outcomes are permanent, (iii) how treatment modifies the outcome, and (iv) how the treatment window changes over time. We take these as the minimal parameters needed to specify a diagnostic scenario. The product structure means that each parameter can, in principle, vary independently of the others: two diagnoses can have identical outcome distributions but different irreversibility structures, or identical treatment effects but different temporal dynamics. Figure 1 depicts this four-factor scenario space.*

### 2.3. Diagnostic Urgency Functions

**Definition** **1.** *A diagnostic urgency function is a function u:D→R. It induces a total preorder ≿ on D via $d≿d^{\prime }$ iff $u\left(d\right)\geq u\left(d^{\prime }\right)$.*

The preorder ≿ satisfies completeness and transitivity by construction (any real-valued function induces a complete, transitive ordering). Continuity is imposed separately as Axiom A6.

**Definition** **2.** *Let $P,P^{\prime }\in \Delta \left(\Omega \right)$. We say P severity-dominates $P^{\prime }$ (written $P{≿}_{SD}P^{\prime }$) if for every threshold k∈{1,…,K}:*

$$\sum_{j=1}^{k}P\left(\omega_{j}\right)\geq \sum_{j=1}^{k}P^{\prime }\left(\omega_{j}\right)$$

This is first-order stochastic dominance with respect to the severity ordering on Ω. In plain terms, *P* severity-dominates $P^{\prime }$ when *P* has worse outcomes in every scenario. The dominance is strict if the inequality is strict for at least one *k*.

**Definition** **3.** *The irreversibility index is the product $\iota \left(C\right)=c\cdot w_{c}\in \left[0,1\right]$.*

**Definition** **4.** *The preventable severity benefit is a continuous function $\beta :E\to R_{\geq 0}$ that measures the expected severity avoided by timely treatment:*

$$\beta \left(E\right)=\max\left(0,\;\sum_{k=1}^{K}s\left(\omega_{k}\right)P^{\mathrm{untx}}\left(\omega_{k}\right)-\sum_{k=1}^{K}s\left(\omega_{k}\right)P^{\mathrm{tx}}\left(\omega_{k}\right)\right)$$

This directional definition gives urgency credit only to expected harm prevented. A treatment that shifts probability from death to recovery receives more credit than one that shifts probability from death to severe disability, and a harmful shift does not increase urgency.

**Definition** **5.** *The time-sensitivity transform is the function σ:T→[0,1] defined by σ(h)=1/(1+h), where h is the treatment half-life in hours and σ(∞)=0.*

### 2.4. Prevalence Independence

Urgency is defined conditional on the diagnosis being present. The prior probability of a diagnosis (its prevalence or pre-test probability) governs which diagnoses to test for and in what order. This is a Bayesian question. Urgency measures the cost of failing to identify a diagnosis that is in fact present. These are distinct questions, and we separate them by construction. The urgency function u(d) takes as input the clinical profile of a diagnosis, not its probability. Section 7.4 makes the integration of urgency with pre-test probability explicit as a product rule and identifies it as the natural bridge from urgency to a complete prioritization decision.

## 3. The Axiom System

### 3.1. Structural Motivation

The axiom system consists of two classes: *sensitivity axioms* and *structural axioms*.

The sensitivity axioms (A1–A4) assert that the urgency function *u* is responsive to each of the four factors of the diagnostic scenario space D. Each axiom asserts monotonicity with respect to one factor while holding the others fixed. There are exactly four such axioms because D has exactly four factors. Adding a fifth sensitivity axiom would require a fifth factor of D. Removing one would allow urgency functions that are insensitive to a clinically relevant dimension.

The structural axioms (A5–A6) make the representation theorem possible. A5 (coordinate independence) is the key structural assumption and corresponds to preferential independence in multiattribute utility theory [22]. It means that a ranking on one dimension does not reverse when the shared values on other dimensions change. A6 (continuity) rules out sudden jumps in urgency from arbitrarily small parameter changes.

The total axiom count (6 = 4 + 2) is thus determined by the structure: one sensitivity axiom per factor of D, plus two structural axioms.

### 3.2. Sensitivity Axioms

**Axiom 1 (A1)** (Severity Dominance)**.** *If $P_{A}{≿}_{SD}P_{B}$ strictly, then for all diagnostic scenarios $d_{A}=\left(P_{A},C,E,h\right)$ and $d_{B}=\left(P_{B},C,E,h\right)$ sharing the same irreversibility, treatment effect, and temporal parameters:*

$$u\left(d_{A}\right)>u\left(d_{B}\right)$$

*Interpretation.* A1 asserts that worse outcome distributions imply higher urgency, all else equal. This is the foundational clinical principle: diagnoses with more severe consequences when missed are more urgent to evaluate. No physician would prioritize a diagnosis with uniformly better outcomes over one with uniformly worse outcomes, given identical irreversibility, treatment effect, and temporal dynamics.

*Necessity.* Without A1, a function u(d)=ι(C)+β(E)+σ(h) (one that ignores outcome severity entirely) would be a valid urgency function. Such a function would assign equal urgency to subarachnoid hemorrhage and tension headache whenever their irreversibility, treatability, and time-sensitivity parameters coincide.

**Axiom 2 (A2)** (Irreversibility Sensitivity)**.** *For diagnostic scenarios differing only in irreversibility parameters: if $\iota \left(C_{A}\right)>\iota \left(C_{B}\right)$, then $u\left(d_{A}\right)>u\left(d_{B}\right)$.*

*Interpretation.* Among diagnoses with identical outcome distributions, treatment effects, and temporal dynamics, those with higher probability of permanent health states are more urgent. Permanence represents an informational boundary: once a health state is permanent, no future intervention can alter it.

*Necessity.* Without A2, urgency would depend only on acute severity and would not distinguish between a diagnosis causing temporary total disability and one causing permanent total disability of equal acute severity. This collapses a clinically critical distinction.

**Axiom 3 (A3)** (Treatability Relevance)**.** *For diagnostic scenarios differing only in treatment effect: if $\beta \left(E_{A}\right)>\beta \left(E_{B}\right)$, then $u\left(d_{A}\right)>u\left(d_{B}\right)$.*

*Interpretation.* A diagnosis where timely identification enables substantial outcome improvement is more urgent than one where identification changes nothing, all else equal. Urgency arises from the actionability of the diagnostic information: the gap between what happens when the diagnosis is missed and what happens when it is made.

*Necessity.* Without A3, a uniformly fatal, untreatable condition and a uniformly fatal, perfectly treatable condition would have equal urgency. The purpose of diagnosis is to enable treatment. A function insensitive to treatability conflates the tragic with the actionable.

**Axiom 4 (A4)** (Temporal Monotonicity)**.** *For diagnostic scenarios differing only in temporal half-life: if $\sigma \left(h_{A}\right)>\sigma \left(h_{B}\right)$ (equivalently, $h_{A}<h_{B}$: shorter treatment half-life), then $u\left(d_{A}\right)>u\left(d_{B}\right)$.*

*Interpretation.* Diagnoses with rapidly closing treatment windows are more urgent than those treatable at any time, all else equal. This captures the difference between ventricular fibrillation (defibrillation window: minutes) and chronic subdural hematoma (surgical window: weeks).

*Necessity.* Without A4, urgency would be insensitive to time pressure, equating the urgency of conditions with minutes-long treatment windows and those treatable indefinitely.

### 3.3. Structural Axioms

**Axiom 5 (A5)** (Coordinate Independence)**.** *For every nonempty proper subset of factors A⊂{1,2,3,4}, the preference between two common configurations on A does not depend on the shared level of the complementary factors.*

Formally, writing $x_{A}$ for the coordinates in *A* and $y_{-A}$ for the complementary coordinates, for all common levels $y_{-A}$ and $y_{-A}^{\prime }$:

$$u\left(x_{A},y_{-A}\right)\geq u\left(x_{A}^{\prime },y_{-A}\right)\Leftrightarrow u\left(x_{A},y_{-A}^{\prime }\right)\geq u\left(x_{A}^{\prime },y_{-A}^{\prime }\right)$$

The single-factor case is the clinically most intuitive instance; the all-subset formulation is the standard condition used for additive representation.

*Interpretation.* Rankings within any block of dimensions do not reverse when the shared levels of the remaining dimensions change. If diagnosis A has worse outcome trajectory than B when both share irreversibility level $C_{0}$, then A still has worse outcome trajectory when both share a different irreversibility level $C_{1}$; similarly, the joint ranking of trajectory-and-treatability profiles is not reversed by changing a shared time-sensitivity level.

Coordinate independence is a no-reversal condition: rankings on a subset of dimensions cannot flip depending on the level of the remaining dimensions. It is an ordinal condition on the direction of a preference, and it does not require the absence of statistical interaction in the magnitude of an outcome (Section 6.3). Together with continuity on the connected product domain, it yields the additive form in Theorem 1, up to a strictly increasing transformation φ.

*Relationship to decision theory.* Coordinate independence is the condition used in Debreu’s [21] additive representation theorem on connected product spaces and corresponds to Keeney and Raiffa’s [22] preferential independence condition in multiattribute utility theory; recent work continues to consolidate the continuity and solvability postulates underlying such representations [24]. We use it as a normative separability condition: it says that the sign of a ranking on one block of attributes should not reverse solely because the shared value of another block changes. It does not assert that the clinical magnitude of each attribute is constant across all contexts.

*Clinical justification.* The all-subset condition is a formal strengthening of the familiar single-dimension no-reversal idea. To make the assumption concrete, consider the six pairwise cases: would a clinician ever reverse the ranking on factor *i* depending on the shared level of factor *j*? The tests are summarized in Table 1.

*Concrete example.* Consider two diagnoses sharing identical treatability (high preventable severity benefit). Diagnosis A has a 2 h treatment window; diagnosis B has a 48 h window. A is more urgent regardless of their shared treatability level. Now consider two diagnoses with identical time-sensitivity but one is highly treatable and the other is not. The treatable one is more urgent regardless of the shared time pressure. The ranking within each dimension is preserved regardless of where the other dimensions are set.

*Limitation.* When one dimension takes extreme values, the practical significance of distinctions on other dimensions may diminish (e.g., when both conditions are certainly fatal, treatability differences become less meaningful clinically). This is not a violation of coordinate independence. The ranking is preserved; only its practical impact changes. We test for coordinate independence violations empirically in Section 6.3.

**Axiom 6 (A6)** (Continuity)**.** *The function u:D→R is continuous with respect to the product topology on D (the natural notion of “closeness” on the combined space) and the standard topology on R.*

*Interpretation.* Small perturbations in any diagnostic parameter produce small changes in urgency. There are no discontinuities where an infinitesimal change in disease parameters causes a finite jump in priority.

*Necessity.* Without continuity, pathological step functions are admitted: e.g., u(d)=1[mortality>0.50], which assigns binary urgency regardless of all other parameters and creates clinically meaningless discontinuities.

### 3.4. Relationship to Decision Theory

The axiom set parallels preferential independence in Keeney and Raiffa [22]. Debreu’s representation theorem [21] provides the mathematical engine: coordinate independence and continuity on a connected product space yield an additive representation. Our contribution is the diagnostic scenario space D, the clinical meaning of the axioms, and the empirical comparison with patient outcomes.

## 4. Main Result

**Theorem 1** (Representation)**.** *Let u:D→R satisfy Axioms A1–A6 and the regularity conditions stated in Supplement S2: complete and transitive induced ordering, connected product domain, continuous upper and lower contour sets, all-subset coordinate independence, nontriviality, and at least three essential coordinates. Then there exist continuous functions $T:\Delta^{\circ }\left(\Omega \right)\to R$, I:C→R, R:E→R, S:T→R such that:*

u(d)=φ(T(P)+I(C)+R(E)+S(h))

*for some continuous, strictly increasing φ:R→R, where T is increasing under severity dominance, I is increasing in the irreversibility index, R is increasing in preventable severity benefit, and S is increasing in the decay rate.*

**Full proof in** **Supplement S2.** **Step 1** (Topological precondition). $D=\Delta^{\circ }\left(\Omega \right)\times C\times E\times T$ is a product of connected spaces. $\Delta^{\circ }\left(\Omega \right)$ is the interior of the simplex (a convex open subset, hence connected); $C={\left[0,1\right]}^{2}$ is connected; E is a product of simplices; T=[0,∞] with the order topology is connected. The product of connected spaces is connected (Munkres [23], Theorem 23.6).

**Step 2** (Debreu’s theorem). Axiom A5 provides coordinate independence; Axiom A6 provides continuity. Axioms A1–A4 supply nontriviality and essentiality of the four coordinates. The remaining topological and regularity premises are listed and checked in Supplement S2. Debreu’s [21] Theorem 3, as stated in Wakker [25], Theorem III.6.6, then yields a continuous additive representation $V\left(d\right)=v_{1}\left(P\right)+v_{2}\left(C\right)+v_{3}\left(E\right)+v_{4}\left(h\right)$ representing the same preference ordering as *u*.

**Step 3** (Monotonicity). Axioms A1–A4 each force the corresponding component to be monotone. For A1: fixing (C,E,h) and using the additive structure, $u\left(d_{A}\right)>u\left(d_{B}\right)$ implies $v_{1}\left(P_{A}\right)>v_{1}\left(P_{B}\right)$ by cancellation. The argument is symmetric for A2–A4.

**Step 4** (Identification). Set $T=v_{1}$, $I=v_{2}$, $R=v_{3}$, $S=v_{4}$. Since *u* and *V* represent the same ordering, u=φ∘V for some continuous, strictly increasing φ. □

For a fixed preference ordering represented by *u*, the additive representation is unique up to a common positive affine transformation of the component functions (Supplement S3). This uniqueness is not a claim that Axioms A1–A6 alone determine a single ordering across all possible urgency functions. The equal-weight choice in the construction (Section 5) is a normalization convention, not a consequence of the axiom set.

## 5. An Explicit Construction

### 5.1. Purpose

Theorem 1 establishes that any urgency function satisfying the axioms admits an additive representation across four clinical attributes. This section gives one concrete construction satisfying all six axioms. This construction verifies that the axiom set is feasible; it is not the only possible scoring rule.

### 5.2. Component Functions

**Outcome Trajectory.** Expected missed-diagnosis severity:

$$T_{\mathrm{sev}}\left(P\right)=\sum_{k=1}^{K}p_{k}\cdot s\left(\omega_{k}\right)$$

This construction is monotone under severity dominance: if *P* shifts probability mass toward more severe outcomes, expected severity increases. It is the axiom-verifying trajectory component and the primary trajectory score used throughout the empirical analyses (Section 6). Separately, and only as exploratory enrichment, we also report a severity-weighted entropy score,

$$H_{s}\left(P\right)=-\sum_{k=1}^{K}p_{k}\cdot s\left(\omega_{k}\right)\cdot \log_{2}\left(p_{k}\right),$$

a descriptive measure of severity-weighted outcome dispersion. $H_{s}$ is useful descriptively but, as detailed in Section 5.4 and Supplement S4a, satisfies A1 only on a characterized sub-region of the simplex, not globally; it is therefore not the axiom-verifying trajectory component and is never used as the primary urgency score.

**Irreversibility.** Expected permanent disability:

$$I\left(C\right)=c\cdot w_{c}$$

**Treatability.** Directional preventable expected severity:

$$R\left(E\right)=\beta \left(E\right)=\max\left(0,\;E\left[s\left(Y_{\mathrm{untx}}\right)\right]-E\left[s\left(Y_{\mathrm{tx}}\right)\right]\right)$$

This is the expected severity prevented by timely treatment (Definition 4). It is directional: benefit increases urgency, while harmful or nonbeneficial shifts do not receive positive urgency credit.

**Time-Sensitivity.** Hyperbolic decay function:

$$S\left(h\right)=\frac{1}{1+h}$$

where *h* is the treatment half-life in hours.

### 5.3. Normalization and Aggregation

For the empirical analysis, each measured component is min-max normalized to [0,1] across the condition set. The primary theorem-aligned composite urgency score uses equal weights and the expected-severity trajectory component $T_{\mathrm{sev}}$:

$$u\left(d\right)=\frac{1}{4}\left[T_{\mathrm{sev}}\left(d\right)+I\left(d\right)+R\left(d\right)+S\left(d\right)\right]$$

The entropy-enriched comparator used in the exploratory analysis replaces $T_{\mathrm{sev}}$ with $H_{s}$ in the same equal-weight aggregation, holding the irreversibility, treatability, and time-sensitivity components fixed; the two composites therefore differ only in the trajectory term. Equal weighting is a reasonable default: absent prior evidence favoring one dimension, equal weights encode minimal assumptions about relative importance (the principle of insufficient reason). The relative weighting is a normalization convention, not a consequence of the axiom set. Sensitivity analysis (Section 6.4) demonstrates robustness across 231 weight combinations.

**Remark** **4.** *The theoretical urgency function u maps any diagnostic scenario in D to a real number. The additive form T+I+R+S is the theoretical object. For the empirical study in Section 6, we apply component-wise min-max normalization across the 37 conditions studied, producing scores in [0,1] for interpretability. This is a scoring convention, not a theorem-level invariance claim: rescaling components separately can change tradeoffs between dimensions and therefore can change some rankings. The normalized scores are reported in Supplementary Table S1.*

### 5.4. Axiom Verification

We verify that the construction satisfies each axiom:

**A1** (Severity Dominance). $T_{\mathrm{sev}}\left(P\right)=\sum_{k}p_{k}s\left(\omega_{k}\right)$ is strictly increasing under strict severity dominance because first-order stochastic dominance with respect to the severity ordering increases the expectation of every strictly increasing severity score. ✓

**Scope of the entropy score.** The severity-weighted entropy score $H_{s}\left(P\right)$ is a descriptive trajectory-dispersion measure, not the axiom-verifying component $T_{\mathrm{sev}}$. It does not satisfy A1 on the full simplex: when probability mass shifts toward a more severe but already high-probability outcome, the dispersion term can fall and $H_{s}$ can decrease. Supplement S4a characterizes this exactly; the region on which $H_{s}$ respects A1 is a strict subset of the simplex interior—$H_{s}$ can violate A1 at strictly interior, non-degenerate distributions—so restricting the domain to $\Delta^{\circ }\left(\Omega \right)$ does not by itself secure A1 for $H_{s}$. Writing $g\left(p\right)=-\log_{2}p-1/\ln2$, an infinitesimal severity-increasing transfer from a less-severe state *ℓ* to a more-severe state *j* changes $H_{s}$ by $\varepsilon [\;s_{j}g\left(p_{j}\right)-s_{\ell }g\left(p_{\ell }\right)\;]$, so $H_{s}$ respects A1 precisely on the region $R=\{P:s_{j}g\left(p_{j}\right)\geq s_{\ell }g\left(p_{\ell }\right)\;\mathrm{whenever}\;s_{j}>s_{\ell }\}$. In the two-state severe/benign case the boundary is $p_{\mathrm{severe}}=1/e\approx 0.368$: below it, increasing severity raises $H_{s}$; above it, $H_{s}$ turns over and falls. By contrast $T_{\mathrm{sev}}$ has constant gradient $s_{k}$ and satisfies A1 globally. The two trajectory scores and their roles are summarized in Table 2.

**A2** (Irreversibility Sensitivity). $I\left(C\right)=c\cdot w_{c}$ is strictly increasing when either factor increases and the other is positive. ✓

**A3** (Treatability Relevance). R(E) is increasing in directional preventable expected severity by construction. ✓

**A4** (Temporal Monotonicity). S(h)=1/(1+h) is strictly decreasing in *h*, hence strictly increasing in time-sensitivity. ✓

**A5** (Coordinate Independence). Each component depends on a single factor of D. The additive structure ensures that rankings on any subset of dimensions are independent of the shared levels of the remaining dimensions. ✓

**A6** (Continuity). Each component function is continuous on its domain. The composite is a finite sum of continuous functions. ✓

## 6. Empirical Criterion-Validity Analyses

### 6.1. Study Design

We report three empirical checks, all anchored on the theorem-aligned score. First, we compare the primary theorem-aligned score with DALY-derived condition weights across the 42-condition registry. Second, we report a retrospective MIMIC-IV (Medical Information Mart for Intensive Care, v2.2) condition-level criterion-validity analysis relating the theorem-aligned score to observed in-hospital outcomes, with the entropy-enriched comparator reported alongside it as exploratory enrichment. Third, we assess whether the score discriminates higher- from lower-acuity conditions (Section 6.3). MIMIC-IV is a single-center critical-care and hospital database from Beth Israel Deaconess Medical Center; it is not a prospective emergency-department differential-diagnosis dataset, and we frame the MIMIC analysis as condition-level criterion validity, not as an ED-workflow simulation. The conditions enter MIMIC-IV through emergency-department presentation, and the cohort retains ED-module timestamps (ED registration and disposition), which partially addresses the concern that the data are unrelated to the ED; nonetheless it remains a retrospective inpatient dataset rather than a prospective ED differential-diagnosis cohort. From an initial registry of 42 emergency conditions across 7 chief complaint clusters (chest pain, dizziness, headache, abdominal pain, dyspnea, syncope, fever; Supplementary Table S1), 37 conditions with sufficient MIMIC-IV representation (N≥50 admissions) were included in the MIMIC analysis, comprising 180,796 admissions from 57,567 unique patients. Five low-acuity conditions (BPPV, vestibular neuritis, tension headache, MSK chest pain, UTI) were excluded from the MIMIC outcome analysis because MIMIC-IV underrepresents benign outpatient diagnoses; these five conditions are retained, with their literature-derived scores, in the discrimination analysis of Section 6.3, where they serve as the low-acuity anchor. We derived urgency dimension scores for each condition from published clinical literature (GBD 2019 disability weights, Cochrane reviews, published case series), independent of MIMIC-IV outcome data. The validation design is condition-level: we test whether literature-derived urgency orderings are associated with observed outcomes.

### 6.2. Primary Result

*Condition-level criterion validity.* The primary theorem-aligned score correlates strongly with DALY-derived condition weights across the 42-condition registry (r=0.934, ρ=0.947). This supports the face and criterion validity of the expected-severity construction, but DALY is an external severity anchor rather than a direct measure of real-time diagnostic prioritization.

In the retrospective MIMIC-IV analysis, the theorem-aligned score correlates with condition-level in-hospital mortality (r=0.684, 95% CI [0.494, 0.837], p<0.001, n=37) and ICU admission rate (r=0.637, 95% CI [0.453, 0.839], p<0.001). It yields a point estimate comparable to published case fatality rate alone (r=0.633, 95% CI [0.423, 0.840]). The entropy-enriched comparator, reported separately as exploratory enrichment, correlates with mortality at a somewhat higher point estimate (r=0.747, 95% CI [0.591, 0.861]) and ICU admission at r=0.750; however, adding $H_{s}$ to the theorem-aligned score in a nested condition-level model improves fit only modestly and non-significantly ($\Delta R^{2}=0.04$, p=0.11, ΔAIC=−0.8). We therefore anchor the empirical claim on the theorem-aligned $T_{\mathrm{sev}}$ score, whose ordering validates the axioms, and treat the entropy enrichment as suggestive rather than confirmatory. The modest drop in the mortality point estimate (0.747→0.684) is the expected cost of using the theorem-valid score, which remains strongly and significantly associated with outcomes. The condition-level results are reported in Table 3.

**Figure 2:**
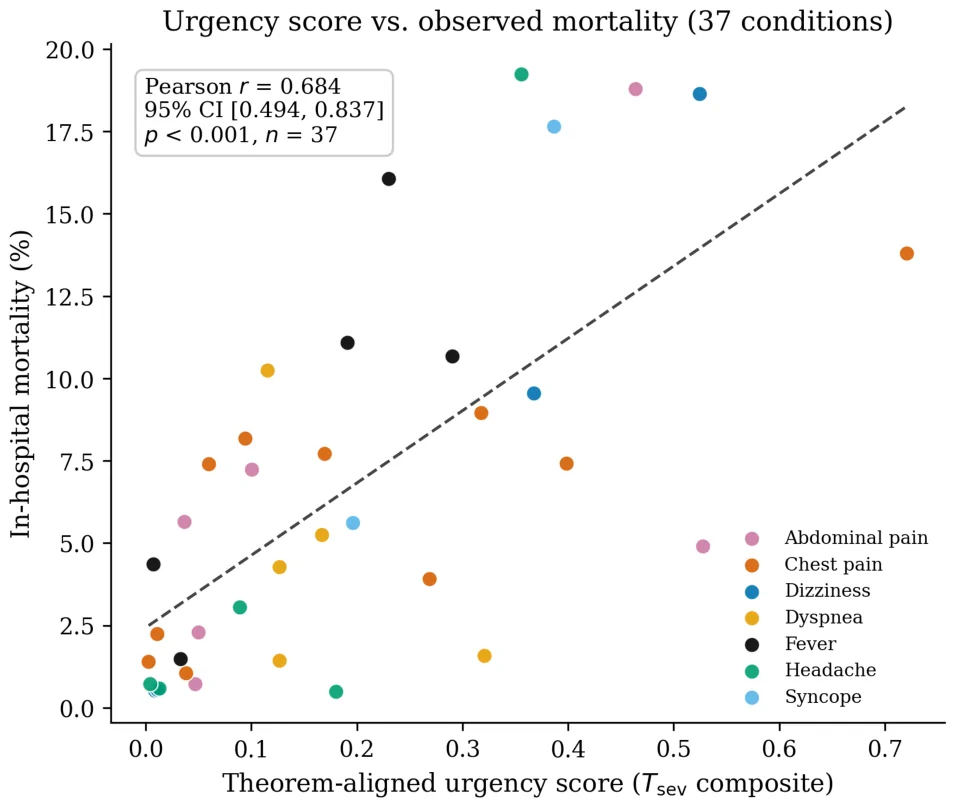
Theorem-aligned urgency score ($T_{\mathrm{sev}}$ composite) versus observed in-hospital mortality for 37 emergency conditions (Pearson r=0.684, 95% CI [0.494, 0.837]). Points are colored by chief-complaint cluster; the dashed line is the ordinary-least-squares fit.

*Patient-level supportive analysis.* At the individual admission level (N=180,796), a GEE logistic regression with exchangeable within-condition clustering, adjusted for age and sex, yields OR =1.88 per IQR increase in the theorem-aligned urgency score (95% CI 1.52–2.34, p<0.001). Because urgency was assigned at the condition level, patient records sharing the same condition also share the same urgency exposure. These patient-level results are therefore supportive and hypothesis-generating; the condition-level analysis is the primary empirical test. Figure 2 displays the condition-level mortality association.

### 6.3. Urgent Versus Non-Urgent Discrimination

A central clinical claim is that the score separates conditions that must not be missed from benign ones. We tested this directly across the full 42-condition registry, including the five low-acuity conditions excluded from the MIMIC outcome analysis. Each condition was assigned an acuity reference label, emergent (an immediate threat to life, limb, or major organ if confirmation or exclusion is delayed beyond hours; ESI level 1–2 equivalent) versus lower-acuity, by author clinical consensus and independently of the urgency score and of MIMIC outcomes; the full labeling (28 emergent, 14 lower-acuity) is listed in Supplementary Table S3.

**Figure 3:**
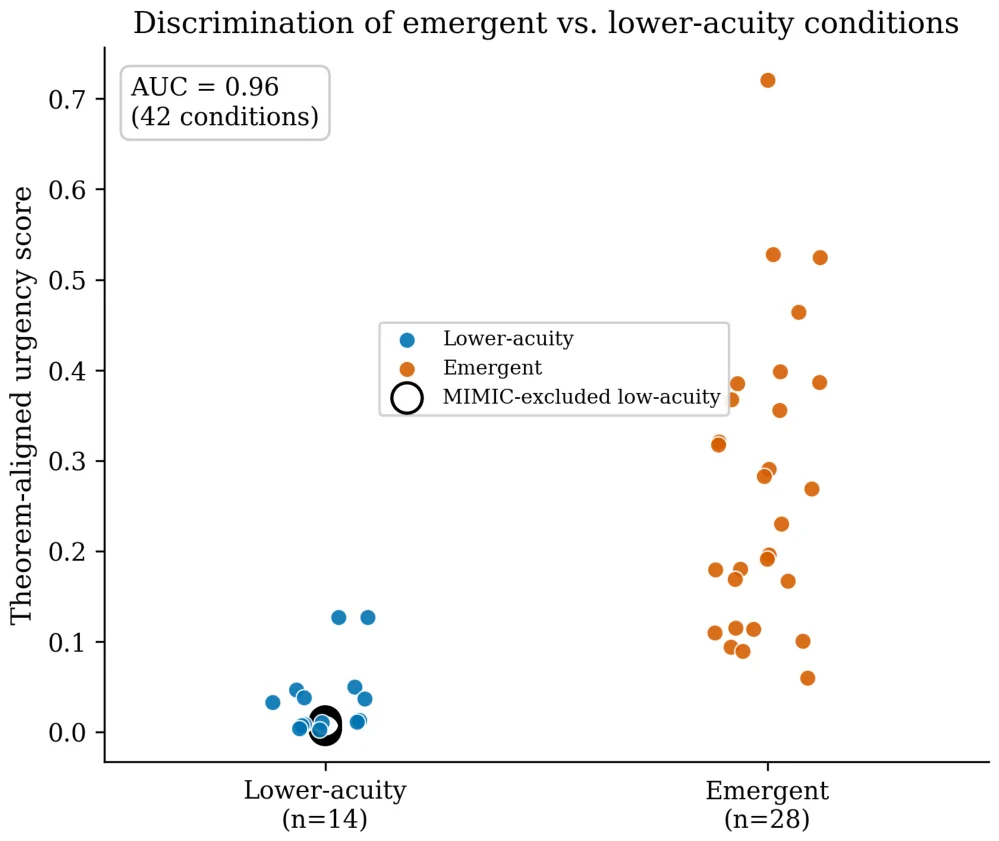
Theorem-aligned urgency score by acuity class for the full 42-condition registry (28 emergent versus 14 lower-acuity, labeled by author clinical consensus independently of the score). The five low-acuity conditions excluded from the MIMIC outcome analysis are circled; all fall at the bottom of the distribution. Area under the ROC curve AUC =0.96.

The theorem-aligned score discriminates the two classes with area under the ROC curve AUC =0.96 (entropy-enriched comparator AUC =0.98). The result is robust to the labeling: flipping any single boundary condition (e.g., appendicitis, cholecystitis, severe pneumonia) between classes leaves AUC in the range 0.95–0.98. As an assumption-free check of rank separation, the five low-acuity conditions excluded from the MIMIC analysis (MSK chest pain, tension headache, UTI, BPPV, vestibular neuritis) all fall in the bottom 14% of the 42-condition score distribution (percentile ranks 2–14%), cleanly separated from the emergent conditions. This is the discrimination the framework requires, and it does not rely on MIMIC outcomes; established ED triage systems and machine-learning acuity models report comparable or lower discrimination on related tasks [11,12]. Figure 3 shows the score distribution by acuity class.

### 6.4. Dimensionality and Interaction Support

The representation theorem is a mathematical result; the following analyses provide supportive empirical evidence for the construction, not validation of the theorem itself.

*Factor analysis on outcome measures.* Factor analysis on the 34×4 condition-by-outcome matrix (34 conditions with N≥200; 3 conditions excluded for insufficient sample size) produced eigenvalues of 2.985, 0.682, 0.220, and 0.113. By the Kaiser criterion, one factor dominates. This reflects the correlation among hospital outcome measures: mortality, ICU admission, and length of stay track the same underlying severity. It does not reflect the urgency dimension structure.

*PCA on urgency scores.* PCA on the four urgency dimension scores yielded eigenvalues of 1.868, 1.311, 0.517, and 0.305. Two components exceed the Kaiser criterion, reflecting empirical correlation between dimensions, particularly trajectory and irreversibility ($r_{s}=0.787$). This correlation is a genuine clinical pattern (conditions with severe trajectories also tend to cause permanent harm) and does not indicate structural redundancy. Two components above Kaiser does not argue for collapsing the framework to two factors: coordinate independence (A5) is an ordinal no-reversal condition, not a claim of statistical orthogonality, and correlated dimensions can remain jointly necessary for the clinical decision. Two conditions can sit at nearly identical positions on the two retained principal components yet diverge on a third dimension in a way that changes clinical action; for example, two conditions with comparable trajectory and irreversibility but very different time-sensitivity (a wide versus a minutes-long treatment window) demand different door-to-treatment targets despite their proximity on the retained components.

*Dimension-removal analysis.* Removing each dimension from the theorem-aligned composite changes its correlation with condition-level mortality as follows: dropping irreversibility reduces it most (Δr=−0.093), dropping the expected-severity trajectory reduces it by Δr=−0.054, while dropping treatability or time-sensitivity slightly increases the mortality correlation (Δr=+0.029 and +0.027). The latter is expected and clinically meaningful rather than evidence against those dimensions: in-hospital mortality is a treatment-confounded proxy for urgency. A highly treatable, time-critical condition has low observed mortality precisely because it is treated promptly, so treatability and time-sensitivity contribute little to predicting raw mortality while remaining essential to the urgency construct and to the DALY-anchored target (against which the full four-dimension composite reaches r=0.934). This is why the framework is anchored on the theorem and the DALY comparison rather than on mortality alone.

*Empirical stress test for coordinate independence (A5).* For each of the 6 dimension pairs, conditions were split at the median on one dimension and the sign of the association between the other dimension, and mortality was examined in each subgroup. We observed no sign reversals (0/6). A5 is an ordinal condition, and this sign-preservation result is the empirically relevant test for it.

*Interaction analysis and the additive approximation.* Because A5 constrains only the direction of rankings, we separately examined whether the magnitude of outcomes shows dimension interactions. Adding all six pairwise interaction terms to the condition-level model raises the explained variance from $R^{2}=0.575$ to $R^{2}=0.833$ (joint $F_{6,26}=6.67$, p<0.001), and five of the six pairwise interactions are individually significant after FDR correction (e.g., trajectory × time, t=4.60, q<0.001). These interactions describe magnitude modulation, not rank reversal, and so are fully consistent with A5; the 0/6 sign-reversal result above is the test that bears on the axiom. In a nested comparison the interaction model improves in-sample fit (adjusted $R^{2}$0.564→0.662), but with six additional parameters estimated on 37 conditions this is exploratory and prone to overfitting. We therefore retain the additive model as the parsimonious, interpretable, theorem-grounded representation, while noting that a non-additive (synergy) representation theory is a principled direction for future work. Two clinical exemplars motivate it: aortic dissection (high irreversibility combined with a narrow treatment window) and mesenteric ischemia (severe trajectory and irreversibility under acute time pressure) are conditions where a multiplicative trajectory–time or irreversibility–time term is clinically plausible.

### 6.5. Robustness

Across 231 weight combinations (varying each dimension weight from 0.10 to 0.40 in increments of 0.05, constrained to sum to 1), all Pearson correlations of the composite with mortality exceeded r=0.50. This suggests that the MIMIC mortality association is not an artifact of the equal-weight convention.

*Severity-granularity sensitivity.* We tested whether the discrete five-state outcome model drives the results by recomputing the trajectory components and the composite under alternative severity granularities: the baseline K=5 partition, perturbed severity anchors (shifting the intermediate severity weights within plausible GBD ranges), a finer K=10 mean-preserving subdivision, and a near-continuous K=20 scheme. The condition ranking is essentially invariant: the Spearman rank correlation of the theorem-aligned composite against the baseline ranking is ≥0.999 across all schemes—the K=10 and near-continuous K=20 mean-preserving refinements leave it exactly unchanged (ρ=1.000), and the small departures (ρ=0.999) arise only from the severity-anchor perturbations—while that of the entropy-enriched composite is ≥0.988. Because the expected-severity trajectory is an expectation, it is exactly invariant under any mean-preserving refinement, so the theorem-aligned ranking is essentially independent of outcome granularity; the entropy enrichment is slightly more sensitive, a further reason to treat it as secondary. The four-factor product structure and the condition ranking are therefore robust to the choice of *K*.

**Figure 4:**
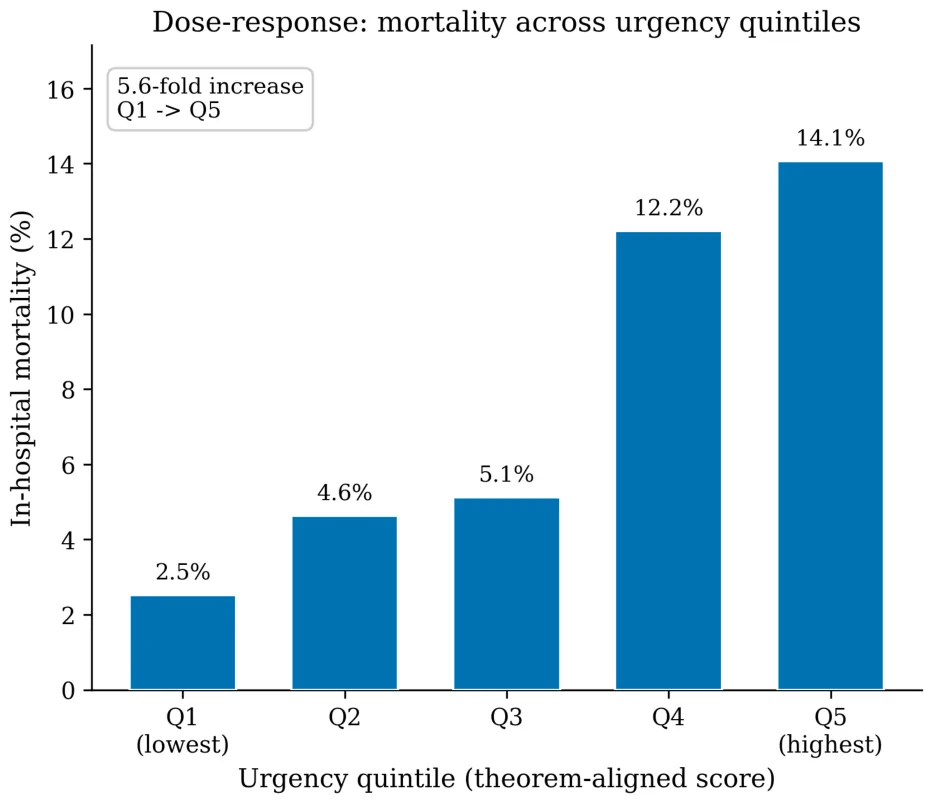
Dose-response of in-hospital mortality across theorem-aligned urgency quintiles: Q1 = 2.5%, Q2 = 4.6%, Q3 = 5.1%, Q4 = 12.2%, Q5 = 14.1%, a 5.6-fold monotone increase from Q1 to Q5.

*Dose-response.* Dose-response across theorem-aligned urgency quintiles shows a monotone gradient: Q1 (lowest urgency) = 2.5% mortality, Q2 = 4.6%, Q3 = 5.1%, Q4 = 12.2%, Q5 (highest urgency) = 14.1%, a 5.6-fold increase from Q1 to Q5. Mortality rises monotonically across all five quintiles, with the steepest step between Q3 and Q4. Observed mortality in the top quintile is nonetheless a treatment-confounded floor rather than a ceiling: several of the highest-urgency conditions (e.g., tension pneumothorax, VT/VF) are rapidly lethal if missed but have relatively low observed mortality because ED teams treat them immediately, so the true urgency gradient is likely steeper than the observed one. Figure 4 shows this monotone relationship.

### 6.6. Limitations of the Empirical Test

Five limitations constrain the empirical analysis. First, the condition set (n=37 in MIMIC-IV) limits statistical power. The within-cluster correlations and the dimension-interaction tests above should be read as exploratory: with chief-complaint clusters of only n=4–10 conditions, power to detect a moderate within-cluster association is roughly 0.2–0.4, so the fact that within-cluster correlations reach significance for only 2 of 6 clusters (chest pain and dizziness) is uninformative about the framework rather than evidence against it. Second, PCA on urgency scores yields approximately two independent axes of variation, not four, because the four urgency dimensions are empirically correlated in this condition set; as discussed in Section 6.3 this reflects clinical co-occurrence, not redundancy. Third, mortality is one proxy for urgency, not its definition; the framework posits that urgency depends on severity, irreversibility, treatability, and time-sensitivity jointly, of which mortality captures primarily the first and is confounded by treatment. Fourth, MIMIC-IV v2.2 is a single-center critical-care and hospital dataset from Beth Israel Deaconess Medical Center, not a real-time ED differential-diagnosis dataset; MIMIC findings should be read as retrospective criterion-validity evidence. Fifth, urgency dimension scores derive from published literature rather than prospective measurement, introducing potential ascertainment bias.

## 7. Discussion

### 7.1. Summary

The representation theorem (Theorem 1) establishes that any urgency function satisfying six clinically motivated axioms and the stated regularity conditions decomposes additively across four dimensions. The theorem-aligned expected-severity construction correlates strongly with DALY-derived condition weights (r=0.934) and with observed in-hospital mortality (r=0.684) and ICU admission (r=0.637) across 37 MIMIC-IV conditions, and it discriminates emergent from lower-acuity conditions (AUC =0.96). A severity-weighted entropy score is reported separately as exploratory enrichment; it tracks mortality at a slightly higher point estimate but adds no statistically significant incremental information over the theorem-aligned score. The key mathematical result is not the list of four attributes, which domain analysis identifies, but the additive aggregation rule, which coordinate independence and continuity support under the regularity assumptions.

### 7.2. Relationship to Clinical Reasoning

The decomposition maps to how clinicians think about diagnostic prioritization. “Rule out the bad things first” becomes: prioritize diagnoses with high expected missed-diagnosis severity, high irreversibility, high preventable treatment benefit, and narrow treatment windows. The four dimensions make implicit clinical reasoning explicit. The additive structure parallels the linear weighted aggregation predominant in healthcare MCDA [19] but supplies the axiomatic justification that most applied MCDA omits, and it complements multi-attribute and entropy-based approaches that have been proposed for triage and clinical decision-making [6,26]. Applications include medical education, clinical decision-support research, and quality measurement.

### 7.3. Limitations

**Coordinate independence.** Axiom A5 is the strongest assumption and should be read as a normative, ordinal separability condition. Dimension interactions in outcome magnitude are real and expected: as reported in Section 6.3, five of six pairwise interaction terms are statistically significant, and a richer interaction model improves in-sample fit. These are magnitude interactions, not rank reversals, and the relevant ordinal test (0/6 sign reversals) supports A5. Nonetheless, systematic synergy is clinically plausible, and a non-additive representation theory is the natural extension; the additive model is retained here as the parsimonious, interpretable approximation grounded in the theorem.

**Five-state outcome model.** The K=5 severity classification is a simplification. Finer-grained outcome states would increase the dimensionality of $\Delta^{\circ }\left(\Omega \right)$ but would not change the four-factor product structure of D; the granularity sensitivity analysis (Section 6.4) confirms that the condition ranking is essentially invariant (ρ≥0.999 for the theorem-aligned score) across K=5, K=10, and near-continuous severity schemes.

**Empirical entropy score.** The severity-weighted entropy score is reported only as an exploratory enrichment. It is not monotone under all severity-dominance comparisons (it respects A1 only on the characterized region R of Supplement S4a), and its incremental predictive value over the theorem-aligned score is not statistically significant. The primary urgency measure throughout is the axiom-verifying expected-severity construction.

**Single-center data.** MIMIC-IV comprises admissions from Beth Israel Deaconess Medical Center. Multi-site validation would strengthen generalizability.

**Limited statistical power.** With 37 conditions and literature-derived scores, dimensionality and interaction tests have limited power and are reported as exploratory.

### 7.4. From Urgency to a Decision Rule

Urgency, as characterized here, is one of two factors a clinician integrates when prioritizing a differential; the other is the pre-test probability that the diagnosis is present. The complete prioritization quantity is their product:

priority(d)∝P(diseasepresent)·u(d),

where u(d) is the conditional urgency this paper characterizes and P(diseasepresent) is the pre-test probability, a Bayesian quantity outside the present scope. The product rule clarifies why urgency must be defined conditional on presence: it is the factor that multiplies probability, not a substitute for it. Consider an acute-headache differential. Subarachnoid hemorrhage has low prevalence but very high urgency; tension-type headache has high prevalence but minimal urgency. Ranking by urgency alone over-prioritizes SAH relative to a workup that also weighs how likely each diagnosis is; ranking by probability alone under-prioritizes SAH; the product reorders the workup to reflect both, capturing the clinical intuition that a rare but catastrophic, treatable diagnosis warrants early exclusion. A complete decision framework therefore couples the urgency function with an estimate of pre-test probability from presentation features and ranks candidates by the product, a multi-attribute prioritization structure consistent with decision-theoretic models proposed for ED triage [26]. Developing and validating that coupled rule, including estimation of P(diseasepresent) from presenting features, is the principal direction for translating this framework toward clinical use.

### 7.5. Future Directions

Beyond the prevalence-coupled decision rule of Section 7.4, future research should evaluate the framework across several general directions: patient-level outcome prediction, dynamic urgency with Bayesian updating during the diagnostic workup, multi-site and prospective emergency-department validation, a non-additive representation theory capturing the synergy interactions observed empirically, integration with clinical decision support systems, and educational applications for medical trainees learning structured diagnostic prioritization. 

## Figures and Tables

**Table 1:** Coordinate-independence reversal tests.

Factor *i*	Factor *j*	Reversal Test	Verdict
*P* (trajectory)	*C* (irreversibility)	“A has worse outcomes than B. Does any shared irreversibility level reverse this?”	No
*P* (trajectory)	*E* (treatability)	“A has worse outcomes than B. Does any shared treatability level reverse this?”	No
*P* (trajectory)	*h* (time)	“A has worse outcomes than B. Does any shared time pressure reverse this?”	No
*C* (irreversibility)	*E* (treatability)	“A causes more permanent harm than B. Does any shared treatability reverse this?”	No
*C* (irreversibility)	*h* (time)	“A causes more permanent harm than B. Does any shared time pressure reverse this?”	No
*E* (treatability)	*h* (time)	“A has better treatment response than B. Does any shared time pressure reverse this?”	No

**Table 2:** Comparison of the theorem-aligned and entropy-enriched trajectory scores.

Score	Definition	Satisfies A1?	Role	Validated Against
$T_{\mathrm{sev}}$ (theorem-aligned)	$\sum_{k}p_{k}s\left(\omega_{k}\right)$	Yes—globally	Axiom-verifying component of Theorem 1; **primary** empirical predictor	DALY weights (r=0.934) and MIMIC mortality (r=0.684)/ICU (r=0.637)
$H_{s}$ (entropy enrichment)	$-\sum_{k}p_{k}s\left(\omega_{k}\right)\log_{2}p_{k}$	Only on $R\subset \Delta^{\circ }\left(\Omega \right)$ (S4a)	Descriptive severity-weighted dispersion; **secondary** enrichment	Incremental over $T_{\mathrm{sev}}$: $\Delta R^{2}=0.04$ (n.s., p=0.11)

**Table 3:** Condition-level criterion-validity results.

Predictor	Outcome	*n*	Pearson *r* [95% CI]	Spearman ρ	*p*
**Theorem-aligned composite ($T_{\mathrm{sev}}$)**	DALY weight	42	0.934 [not bootstrapped]	0.947	<0.001
**Theorem-aligned composite ($T_{\mathrm{sev}}$)**	Mortality	37	0.684 [0.494, 0.837]	0.698	<0.001
**Theorem-aligned composite ($T_{\mathrm{sev}}$)**	ICU rate	37	0.637 [0.453, 0.839]	0.726	<0.001
Entropy-enriched comparator ($H_{s}$)	Mortality	37	0.747 [0.591, 0.861]	0.750	<0.001
Entropy-enriched comparator ($H_{s}$)	ICU rate	37	0.750 [0.624, 0.851]	0.771	<0.001
Irreversibility	ICU rate	37	0.593 [0.229, 0.783]	0.580	<0.001
Treatability	Mortality	37	0.455 [0.233, 0.701]	0.574	0.005
Time-sensitivity	Deterioration ^*a*^	37	0.367 [0.054, 0.626]	0.275	0.025
Published CFR	Mortality	37	0.633 [0.423, 0.840]	0.793	<0.001

^*a*^ Deterioration: proportion of admissions with clinical worsening, defined as transfer to a higher level of care (floor to ICU) or in-hospital cardiac arrest.

## Data Availability

MIMIC-IV data are available through PhysioNet at https://physionet.org/content/mimiciv/ (accessed on 9 July 2026). Analysis code and urgency score definitions are available at https://github.com/HeScience/diagnostic-urgency (accessed on 15 July 2026).

## Supplementary Materials

The following supporting information can be downloaded at: https://www.mdpi.com/article/10.3390/e28080853/s1. *Supplementary Materials: Axioms of Diagnostic Urgency: A Characterization Theorem*, including formal definitions, the full proof of the representation theorem, the uniqueness result, construction verification, and empirical sensitivity analyses.

## Abbreviations

The following abbreviations are used in this manuscript:

APACHE	Acute Physiology and Chronic Health Evaluation
BPPV	Benign paroxysmal positional vertigo
CFR	Case fatality rate
ED	Emergency department
ESI	Emergency Severity Index
GBD	Global Burden of Disease
GEE	Generalized estimating equation
ICU	Intensive care unit
MCDA	Multi-criteria decision analysis
MIMIC-IV	Medical Information Mart for Intensive Care IV
NEWS	National Early Warning Score
PCA	Principal component analysis
SOFA	Sequential Organ Failure Assessment
TBI	Traumatic brain injury
VT/VF	Ventricular tachycardia/ventricular fibrillation
