# Supplementary material for: Axioms of Diagnostic Urgency: A Characterization Theorem
Source: Entropy (Basel). 2026 Jul 31;28(8):853. doi: 10.3390/e28080853 (PMC13512457; doi:10.3390/e28080853)
Supplement: Supplementary file 1 [file entropy-28-00853-s001.zip › Definitions/logo-updates-eps-converted-to.pdf]

Check for updates
