# Supplementary material for: Axioms of Diagnostic Urgency: A Characterization Theorem
Source: Entropy (Basel). 2026 Jul 31;28(8):853. doi: 10.3390/e28080853 (PMC13512457; doi:10.3390/e28080853)
Supplement: Supplementary file 1 [file entropy-28-00853-s001.zip › entropy-4364435-supplementary.pdf]

Supplementary Materials: Axioms of Diagnostic Urgency: A Characterization Theorem

Pedram Safari [1], Amal Mohamed [2] and Shuhan He [2,*]

1. S1. Formal Definitions

Definition 1 (Outcome Space). Let Omega = {o_1, o_2, . . . , o_K} be a finite set of health outcome states, equipped with a severity function s: Omega -> [0, 1] satisfying s(o_1) > s(o_2) > . . . > s(o_K) >= 0. We fix K = 5 with the canonical ordering: o_1 = death (s_1 = 1.00), o_2 = severe disability (s_2 = 0.55), o_3 = moderate disability (s_3 = 0.35), o_4 = mild disability (s_4 = 0.10), o_5 = full recovery (s_5 = 0.00). Let Delta(Omega) denote the standard (K-1)-simplex of probability distributions over Omega, i.e., Delta(Omega) = {P = (p_1, . . . , p_K) : p_k >= 0, sum_{k=1}^{K} p_k = 1}. Let Delta^o(Omega) denote the interior of the simplex, i.e., Delta^o(Omega) = {P in Delta(Omega) : p_k > 0 for all k}.

Definition 2 (Irreversibility Parameters). Let C = [0, 1] x [0, 1] be the irreversibility parameter space, where a pair (c, w_c) in C represents: c = probability of transitioning to a permanent (absorbing) health state if the diagnosis is missed, and w_c = disability weight of that absorbing state.

Definition 3 (Treatment Effect Space). Let E = Delta(Omega) x Delta(Omega) be the treatment effect space. A pair (P^tx, P^untx) in E represents the outcome distributions with and without timely treatment, respectively. The treatment effect is measured by directional preventable expected severity:

```
B(P^tx, P^untx) = max(0, sum_{k=1}^{K} s_k * p_k^untx - sum_{k=1}^{K} s_k * p_k^tx).
```

This definition gives positive urgency credit only to expected harm prevented by treatment. It does not reward a harmful shift in the outcome distribution.

Definition 4 (Temporal Half-Life Space). Let T_H = [0, infinity] be the scalar half-life domain for treatment effectiveness. A value h = 0 denotes immediate loss of treatment benefit. A finite h denotes the time scale over which benefit falls by half. A value h = infinity denotes no measurable time-sensitive loss of benefit over the modeled horizon. We equip T_H with the order topology. It is compact and connected, and the map h -> 1/(1+h), extended by 1/(1+infinity) = 0, is continuous.

Definition 5 (Diagnostic Scenario). A diagnostic scenario is a tuple d = (P, C, E, h) in D, where D = Delta^o(Omega) x C x E x T_H is the product space of all diagnostic scenarios. The domain restriction to the interior Delta^o(Omega) excludes degenerate distributions while preserving the connected product structure used in the representation theorem.

Definition 6 (Diagnostic Urgency Function). A diagnostic urgency function is a mapping u: D -> R from the space of diagnostic scenarios to the real numbers, representing the priority of resolving each diagnosis.

Remark 1 (Topology of D). Each factor space is a connected topological space: Delta^o(Omega) is the interior of the standard simplex in R^K, which is open, convex, and connected; C = [0, 1]^2 is compact and connected; E = Delta(Omega) x Delta(Omega) is a product of simplices, hence connected; T_H = [0, infinity] with the order topology is connected. Therefore D, as a product of connected spaces, is connected.

2. S2. Proof of Theorem 1 (Representation)

Theorem 1 (Representation). Let u: D -> R be a diagnostic urgency function satisfying Axioms A1–A6 and the regularity conditions listed below, where D = Delta^o(Omega) x C x E x T_H. Then there exist continuous functions

```
T: Delta^o(Omega) -> R,  I: C -> R,  R: E -> R,  S: T_H -> R
```

and a continuous, strictly increasing function phi: R -> R such that

```
u(d) = phi(T(P) + I(C) + R(E) + S(h))
```

for all d = (P, C, E, h) in D, where T is increasing under severity dominance, I is increasing in the irreversibility index, R is increasing in preventable severity benefit, and S is increasing in time-sensitivity.

2.1. Representation-Theorem Premise Checklist

The proof uses Debreu's additive representation theorem for connected product spaces, as stated in Wakker (1989), Theorem III.6.6. The required premises are handled as follows.

Debreu/Wakker premise	Source in this paper
Cartesian product domain with at least three factors	D = Delta^o(Omega) x C x E x T_H has four factors
Connected topological factor spaces	Verified in Step 1
Complete and transitive preference ordering	Induced by the real-valued function u
Continuous preference ordering	A6 plus continuity of u
Coordinate independence	A5, stated for every nonempty proper subset of factors
Nontrivial ordering	Any one of A1-A4 gives strict comparisons
At least three essential coordinates	Verified before theorem application from A1-A4

The checklist separates clinical axioms from mathematical regularity conditions. Completeness, transitivity, connectedness, continuity, coordinate independence, nontriviality, and essentiality are the assumptions needed to apply the representation theorem; A1-A4 then identify the monotonic direction of the component functions.

2.2. Proof

We proceed in four steps.

Step 1. D is a connected product space.

We must verify that D = Delta^o(Omega) x C x E x T_H is a connected topological space expressible as a product of connected factors. Each factor is a connected topological space:

(i) Delta^o(Omega) is the interior of the standard (K-1)-simplex in R^K. As an open convex subset of Euclidean space, it is path-connected, hence connected.
(ii) C = [0, 1] x [0, 1] is a product of closed intervals, hence path-connected and connected.
(iii) E = Delta(Omega) x Delta(Omega) is a product of two simplices, each connected, so E is connected.
(iv) T_H = [0, infinity] with the order topology is connected. The endpoint h = infinity represents no measurable loss of treatment benefit over the modeled horizon. It is not itself a decay curve; it is the boundary value of the scalar half-life coordinate. The time-sensitivity transform S(h) = 1/(1+h), with S(infinity) = 0, is continuous at this endpoint.

Since a finite product of connected spaces is connected (Munkres, Theorem 23.6), D is connected. QED (Step 1).

Step 2. Axiom A5 is coordinate independence; A6 is continuity.

Axiom A5 states that the preference ordering >=_u on D is coordinate-independent in the all-subset sense: for every nonempty proper subset of coordinates, preferences between two configurations on that subset are unchanged when the complementary coordinates are replaced by a common alternative level. This is the independence condition used in Debreu-style additive representation theorems.

Axiom A6 states that u is continuous on D. Since u is continuous and represents the preference ordering >=_u (i.e., u(d) >= u(d′) if and only if d >=_u d′), the preference ordering >=_u is continuous in the topological sense: for every d in D, the sets {d′ : d′ >=_u d} and {d′ : d >=_u d′} are closed.

Step 3. Essential coordinates and application of Debreu's Representation Theorem.

Before invoking the theorem, we verify essentiality. A coordinate is essential if varying that coordinate while holding the others fixed can change the induced ordering. A1 makes the outcome coordinate essential. A2 makes the irreversibility coordinate essential. A3 makes the treatment-effect coordinate essential. A4 makes the temporal coordinate essential. Thus all four coordinates are essential, satisfying the "at least three essential coordinates" requirement.

We now invoke the following classical result:

Debreu (1960), Theorem 3 (as stated in Wakker (1989), Theorem III.6.6): Let X = X_1 x X_2 x … x X_n be a Cartesian product of connected topological spaces with at least three essential coordinates. Suppose >= is a continuous, complete, transitive preference ordering on X satisfying coordinate independence for the relevant coordinate subsets. Suppose furthermore that the ordering is nontrivial (there exist x, y in X with x > y). Then there exist continuous functions v_i: X_i -> R, i = 1, …, n, such that for all x, y in X:

x >= y if and only if SUM_{i=1}^{n} v_i(x_i) >= SUM_{i=1}^{n} v_i(y_i).

We verify the premises:

(a) *Product of connected spaces.* Verified in Step 1, with n = 4.
(b) *Complete and transitive ordering.* The function u: D -> R induces a preference ordering >=_u defined by d >=_u d′ iff u(d) >= u(d′). Since R is totally ordered, >=_u is automatically complete and transitive.
(c) *Continuity.* By Axiom A6, u is continuous. This implies that the upper and lower contour sets {d′ : u(d′) >= u(d)} and {d′ : u(d′) <= u(d)} are closed for every d, which is the standard definition of a continuous preference ordering.
(d) *Coordinate independence.* This is precisely Axiom A5.
(e) *Nontriviality.* By Axiom A1, there exist distributions P, P′ in Delta^o(Omega) with P strictly severity-dominating P′. For any fixed (C, E, h), the scenarios d = (P, C, E, h) and d′ = (P′, C, E, h) satisfy u(d) > u(d′). Hence the ordering is nontrivial.

All premises are satisfied. By Debreu's theorem, there exist continuous functions

```
v_1: Delta^o(Omega) -> R,   v_2: C -> R,   v_3: E -> R,   v_4: T_H -> R
```

such that for all d = (P, C, E, h) and d′ = (P′, C′, E′, h′) in D:

```
u(d) >= u(d')  if and only if  v_1(P) + v_2(C) + v_3(E) + v_4(h) >= v_1(P') + v_2(C') + v_3(E') + v_4(h').
```

Define V(d) = v_1(P) + v_2(C) + v_3(E) + v_4(h). Then u and V represent the same preference ordering, so there exists a strictly increasing function phi: R -> R such that u(d) = phi(V(d)) for all d.

Step 4. Monotonicity from A1–A4.

We now use Axioms A1–A4 to establish the monotonicity properties of the component functions.

A1 implies v_1 is increasing in severity dominance. Fix (C, E, h) and consider d = (P, C, E, h) and d′ = (P′, C, E, h) with P strictly severity-dominating P′, both in Delta^o(Omega). By A1, u(d) > u(d′), which implies V(d) > V(d′) (since phi is strictly increasing). Since d and d′ share (C, E, h), v_2(C) + v_3(E) + v_4(h) cancels, yielding v_1(P) > v_1(P′). Hence v_1 is strictly increasing with respect to severity stochastic dominance.

*A2 implies v_2 is increasing in c w_c.** Fix (P, E, h) and consider d = (P, C, E, h) and d′ = (P, C′, E, h) with c * w_c > c′ * w_c′. By A2, u(d) > u(d′), and by the same cancellation argument, v_2(C) > v_2(C′). Hence v_2 is strictly increasing in the product c * w_c.

A3 implies v_3 is increasing in preventable severity benefit. Fix (P, C, h) and consider d = (P, C, E, h) and d′ = (P, C, E′, h) with B(E) > B(E′). By A3, u(d) > u(d′), giving v_3(E) > v_3(E′). Hence v_3 is strictly increasing in directional preventable expected severity.

A4 implies v_4 is decreasing in half-life (increasing in time-sensitivity). Fix (P, C, E) and consider d = (P, C, E, h) and d′ = (P, C, E, h′) with h < h′. By A4, u(d) > u(d′), giving v_4(h) > v_4(h′). Since shorter half-life corresponds to greater time-sensitivity, v_4 is strictly decreasing in h.

Now define:

```
T = v_1,  I = v_2,  R = v_3,  S = v_4.
```

Each component function is continuous (by Debreu's theorem). Therefore:

```
u(d) = phi(v_1(P) + v_2(C) + v_3(E) + v_4(h)) = phi(T(P) + I(C) + R(E) + S(h))
```

where phi is continuous and strictly increasing. The monotonicity properties of T, I, R, S established above complete the identification. QED.

3. S3. Uniqueness for a Fixed Preference Ordering

Proposition S1 (Uniqueness for a fixed ordering). Fix the preference ordering >=_u induced by an urgency function u: D -> R satisfying Axioms A1–A6. Suppose this same ordering admits two representations:

```
u(d) = F(T(P), I(C), R(E), S(h)) = F*(T*(P), I*(C), R*(E), S*(h))
```

both satisfying the conditions of Theorem 1. Then there exist strictly increasing functions phi_1, phi_2, phi_3, phi_4 and a strictly increasing function Phi such that:

```
T* = phi_1 composed with T,   I* = phi_2 composed with I,   R* = phi_3 composed with R,   S* = phi_4 composed with S
```

and

```
F*(phi_1(T), phi_2(I), phi_3(R), phi_4(S)) = Phi(F(T, I, R, S)).
```

In particular, for a fixed represented ordering, the additive component functions are unique up to a common positive affine transformation after choosing an additive scale. This is a representation-uniqueness claim for a given ordering, not a claim that Axioms A1–A6 alone determine a single ordering across all possible urgency functions.

3.1. Proof

By the proof of Theorem 1, each representation arises from an application of Debreu's theorem. Debreu's theorem provides additive representations:

```
V(d) = v_1(P) + v_2(C) + v_3(E) + v_4(h)
```

and a corresponding primed representation:

```
V*(d) = v_1*(P) + v_2*(C) + v_3*(E) + v_4*(h).
```

Both V and V* represent the same preference ordering >=_u.

By the uniqueness clause of Debreu's theorem (Debreu, 1960, Section 5; Wakker, 1989, Theorem III.6.6), if the product space has at least three essential coordinates (coordinates along which the preference ordering is non-degenerate), then the additive representation is unique up to a common positive affine transformation. That is, there exist constants a > 0 and b_1, b_2, b_3, b_4 in R such that:

```
v_i*(x_i) = a * v_i(x_i) + b_i   for all x_i and all i = 1, ..., 4.
```

Verification of essentiality. A coordinate i is essential if there exist values x_i and x_i' in X_i such that v_i(x_i) differs from v_i(x_i'). By the monotonicity results established in Step 4 of the proof of Theorem 1:

- v_1 is strictly increasing in severity dominance (A1), so coordinate 1 is essential (compare any distribution P with P' where P strictly dominates P').
- v_2 is strictly increasing in c * w_c (A2), so coordinate 2 is essential (compare c * w_c = 0 with c * w_c = 1).
- v_3 is strictly increasing in preventable severity benefit (A3), so coordinate 3 is essential (compare B = 0 with B > 0).
- v_4 is strictly increasing in time-sensitivity (A4), so coordinate 4 is essential (compare h = 0 with h = infinity).

Since all four coordinates are essential (and 4 >= 3), Debreu's uniqueness result applies. Therefore v_i* = a * v_i + b_i for each i.

Now, the general representations are:

```
T = v_1,  T* = v_1* = a * v_1 + b_1 = a * T + b_1
```

and similarly for I, R, S. Define phi_1(x) = a * x + b_1 (a positive affine, hence strictly increasing function), and likewise phi_2(x) = a * x + b_2, phi_3(x) = a * x + b_3, phi_4(x) = a * x + b_4.

The additive representations are related by:

```
V*(d) = a * V(d) + (b_1 + b_2 + b_3 + b_4).
```

Since u(d) = phi(V(d)) = phi(*V*(d)) for strictly increasing functions phi and phi*, the aggregation functions F and F* are related by:

```
F*(phi_1(T), phi_2(I), phi_3(R), phi_4(S)) = phi*(a * (T + I + R + S) + b_1 + b_2 + b_3 + b_4)
```

which is a strictly increasing function of u = F(T, I, R, S). Define Phi accordingly.

Corollary (No uniqueness across axiom-satisfying functions). For any fixed preference ordering represented by u, the additive representation is unique up to a common positive affine transformation of the component functions. The axioms do not by themselves determine a unique rank ordering across all possible urgency functions satisfying A1–A6; different monotone component scales or tradeoffs can satisfy the same axioms while ranking cross-dimensional tradeoffs differently. Thus the equal-weight choice in the construction (main text Section 5) is a normalization and modeling convention, not a consequence of the axiom set.

QED.

4. S4. Axiom Verification for the Construction

We verify that the explicit construction satisfies all six axioms. The construction is:

```
T(P) = sum_{k=1}^{K} p_k * s_k                    (expected missed-diagnosis severity)
I(C) = c * w_c                                      (expected permanent disability)
R(E) = max(0, E[s(Y_untx)] - E[s(Y_tx)])          (directional preventable severity)
S(h) = 1 / (1 + h)                                 (hyperbolic transform of half-life)
u(d) = T(P) + I(C) + R(E) + S(h)                   (additive aggregation)
```

4.1. Verification of A1 (Severity Dominance)

We must show: if P severity-dominates P′ in the first-order stochastic dominance sense with respect to the severity ordering, then T(P) >= T(P′), with strict inequality when dominance is strict.

Recall T(P) = sum_k p_k * s_k, the expected severity of the missed-diagnosis outcome distribution. Because the severity weights satisfy s_1 > s_2 > . . . > s_K, first-order stochastic dominance toward more severe outcomes increases the expected severity score. Equivalently, any strict dominance relation can be represented as a finite sequence of probability-mass shifts from less severe states to more severe states; each shift from l to j with j < l changes T by epsilon * (s_j - s_l) > 0. Therefore strict severity dominance implies T(P) > T(P′). QED.

Remark (Entropy score). The main text reports H_s(P) = sum_k p_k * s_k * (-log_2 p_k) as a severity-weighted trajectory-dispersion score, only as exploratory enrichment. H_s(P) is not the axiom-verifying trajectory component: entropy can decrease under severity dominance when probability mass is shifted toward a more severe but already common outcome. Thus H_s should be interpreted as a working empirical score for outcome dispersion weighted by severity, whereas T(P) = E[s] is the construction that verifies A1 and is the primary trajectory score throughout. Section S4a below characterizes exactly the sub-region of the simplex on which H_s does respect A1.

4.2. Verification of A2 (Irreversibility Sensitivity)

We must show: if c * w_c > c′ * w_c′, then I(C) > I(C′).

This is immediate from the definition: I(C) = c * w_c. QED.

4.3. Verification of A3 (Treatability Relevance)

We must show: if B(E) > B(E′), then R(E) > R(E′).

Recall R(E) = B(E) by definition, where B(E) = max(0, E[s(Y_untx)] - E[s(Y_tx)]). Therefore B(E) > B(E′) implies R(E) > R(E′). QED.

Toy example. Suppose untreated disease leads to death with probability 1. If timely treatment leads to full recovery with probability 1, then B = 1.00 - 0.00 = 1.00. If timely treatment instead leads to severe disability with probability 1, then B = 1.00 - 0.55 = 0.45. The recovery-producing treatment is therefore more treatable than the severe-disability-producing treatment. Under an absolute distributional-distance metric, the second case could be incorrectly rewarded as much as or more than the first. The directional definition avoids that error.

4.4. Verification of A4 (Temporal Monotonicity)

We must show: if h < h′, then S(h) > S(h′); and if h = h′, then S(h) = S(h′).

Recall S(h) = 1/(1 + h). We compute:

```
dS/dh = -1/(1 + h)^2 < 0   for all h >= 0.
```

Therefore S is strictly decreasing in h. If h < h′, then S(h) = 1/(1 + h) > 1/(1 + h′) = S(h′). If h = h′, then S(h) = S(h′). QED.

4.5. Verification of A5 (Coordinate Independence / Separability)

We must show: the preference ordering induced by u(d) = T(P) + I(C) + R(E) + S(h) is coordinate-independent for every nonempty proper subset of factors.

Let A be any such subset, and write V_A(x_A) for the sum of the component functions whose coordinates lie in A. Write V_{-A}(y_{-A}) for the sum of the remaining component functions. The additive score can be written:

```
u(x_A, y_{-A}) = V_A(x_A) + V_{-A}(y_{-A}).
```

For any common complementary level y_{-A}, the comparison

```
u(x_A, y_{-A}) >= u(x_A', y_{-A})
```

is equivalent to

```
V_A(x_A) + V_{-A}(y_{-A}) >= V_A(x_A') + V_{-A}(y_{-A}).
```

The common term V_{-A}(y_{-A}) cancels, so this is equivalent to V_A(x_A) >= V_A(x_A'). The same cancellation occurs for any other common complementary level y_{-A}'. Therefore the comparison between x_A and x_A' does not depend on the shared complementary coordinates. Hence the additive construction satisfies A5. QED.

4.6. Verification of A6 (Continuity)

We must show: u(d) = T(P) + I(C) + R(E) + S(h) is continuous on D.

It suffices to show that each component function is continuous, since a finite sum of continuous functions is continuous.

(i) *T(P) = sum_k p_k* s_k is continuous on Delta^o(Omega).* This is a finite linear combination of the coordinate functions p_k, hence continuous.
(ii) *I(C) = c* w_c is continuous on C = [0,1]^2.* The product of two continuous functions on [0,1]^2 is continuous. Immediate.
(iii) *R(E) = max(0, E[s(Y_untx)] - E[s(Y_tx)]) is continuous on E.* Expected severity is a finite linear combination of probabilities, subtraction is continuous, and max(0, x) is continuous. Immediate.
(iv) *S(h) = 1/(1+h) is continuous on T_H.* The function g(h) = 1/(1+h) is continuous on [0, infinity). At h = infinity, we define S = 0, and lim_{h -> infinity} 1/(1+h) = 0, so the extension is continuous.

Therefore u = T + I + R + S is continuous on D. QED.

4.7. S4a. The Simplex Region on Which H_s Respects Axiom A1

The empirical analysis reports a severity-weighted entropy trajectory score $H_s(P) = -\sum_{k=1}^{K} p_k\, s_k \log_2 p_k$ as an exploratory enrichment. Unlike the expected-severity component $T_{\mathrm{sev}}(P) = \sum_k p_k s_k$, H_s does not respect Axiom A1 (severity dominance) on the full simplex. We characterize exactly where it does.

Setup. Let the ordered states have severities $s_1 > \cdots > s_K$ in $[0,1]$, and let $P = (p_1, \ldots, p_K)$ range over the relative interior $\Delta^\circ(\Omega)$. A1 requires that transferring probability mass from a less-severe state ℓ to a more-severe state j (so $s_j > s_\ell$) does not decrease the score.

Proposition S4a. Define $g(p) = -\log_2 p - \frac{1}{\ln 2}$, where $\frac{1}{\ln 2} = \log_2 e \approx 1.442695$. The gradient of H_s is $\partial H_s / \partial p_k = s_k\, g(p_k)$. For the infinitesimal severity-increasing transfer $\mathrm{d}p_j = +\varepsilon$, $\mathrm{d}p_\ell = -\varepsilon$ $(s_j > s_\ell)$,

$$\mathrm{d}H_s \;=\; \varepsilon\big[\, s_j\, g(p_j) - s_\ell\, g(p_\ell) \,\big],$$

so H_s is locally non-decreasing under the transfer, i.e., respects A1 at P, iff

$$s_j\, g(p_j) \;\geq\; s_\ell\, g(p_\ell). \qquad (*)$$

The region on which H_s respects A1 is $\mathcal{R} = \{\, P \in \Delta^\circ(\Omega) : s_j g(p_j) \geq s_\ell g(p_\ell)$ whenever $s_j > s_\ell \,\}$.

Proof. Differentiating $-s_k p_k \log_2 p_k$ gives $\partial H_s/\partial p_k = -s_k(\log_2 p_k + \frac{1}{\ln 2}) = s_k g(p_k)$. The transfer yields $\mathrm{d}H_s = (\partial H_s/\partial p_j)\varepsilon + (\partial H_s/\partial p_\ell)(-\varepsilon) = \varepsilon[s_j g(p_j) - s_\ell g(p_\ell)]$, which is ≥ 0 iff $(*)$. □

Interpretation. The quantity $s_k g(p_k)$ is the severity-weighted surprisal offset of state k. H_s obeys severity dominance exactly where the gaining (more-severe) state carries at least as much severity-weighted surprisal offset as the losing state. Since $g(p)$ is decreasing and changes sign at $p = 1/e$, $(*)$ can fail when the more-severe state is already high-probability (small $g(p_j)$) while the less-severe state is rare (large $g(p_\ell)$): adding mass to an already-likely severe outcome reduces dispersion and can lower H_s.

Two-state boundary. For $K = 2$ with a severe state ($s_j = 1$) and a benign state ($s_\ell = 0$), $(*)$ reduces to $g(p_j) \geq 0$, i.e. $p_j \leq 2^{-1/\ln 2} = e^{-1} \approx 0.368$. Thus shifting mass onto the severe state raises H_s only while $p_{\text{severe}} < 1/e$; beyond $1/e$ the same severity-increasing shift lowers H_s, violating A1. The function $H_s(p_{\text{severe}}) = -p \log_2 p$ attains its maximum at exactly $p = 1/e$, which is the boundary $\partial\mathcal{R}$ in this case.

$\mathcal{R}$ is a strict subset of the interior. The region $\mathcal{R}$ is a property of the score H_s, not of the domain, and it is **not** secured by the restriction of the scenario space to the interior $\Delta^\circ(\Omega)$: H_s violates A1 at many strictly interior, non-degenerate distributions, so excluding boundary distributions does not by itself make H_s comply with severity dominance. For a concrete five-state illustration with $s = (1.0, 0.55, 0.35, 0.10, 0.0)$, take the interior distribution $P = (0.50, 0.20, 0.15, 0.05, 0.10)$ (every coordinate strictly positive) and shift an infinitesimal mass ε from mild disability ($s_4 = 0.10$, $p_4 = 0.05$) to death ($s_1 = 1.0$, $p_1 = 0.50$)—a strict severity-dominance move. Then $\mathrm{d}H_s = \varepsilon[s_1 g(p_1) - s_4 g(p_4)] = \varepsilon[(1.0)(-0.443) - (0.10)(2.879)] = -0.73\,\varepsilon < 0$: making death more likely *lowers* H_s. (Numerically, H_s falls from 0.9207 to 0.9200 under this transfer.) The violation occurs well inside the interior, which is why a separate characterization of $\mathcal{R}$ is required and why the interior domain assumption alone—although appropriate for the representation theorem—does not place H_s in compliance with A1.

Contrast with T_{sev}. The expected-severity component has constant gradient $\partial T_{\text{sev}}/\partial p_k = s_k$, so the same transfer always gives $\mathrm{d}T_{\text{sev}} = \varepsilon(s_j - s_\ell) > 0$: T_{sev} satisfies A1 on all of $\Delta^\circ(\Omega)$. This is why the manuscript anchors the empirical claim on T_{sev} and presents H_s as an enrichment whose axiom-validity is confined to $\mathcal{R}$. The condition $(*)$, the $1/e$ boundary, and the global monotonicity of T_{sev} were verified numerically (100% sign agreement of $(*)$ with exact $\mathrm{d}H_s$ over 15,626 random transfers; boundary $= 1/e$ to 10^{-9}); the verification code is available in the analysis repository.

5. S4b. Severity-Granularity Sensitivity

Because T_{sev} is an expectation, it is invariant under any mean-preserving refinement of the severity partition; the four-factor product structure of $\mathcal{D}$ and the condition ranking therefore do not depend on the choice of K (main text Remark 1). We confirmed this empirically by recomputing the trajectory components and the composite under the baseline $K = 5$ partition, perturbed intermediate severity anchors (within plausible GBD ranges), a finer $K = 10$ mean-preserving subdivision, and a near-continuous $K = 20$ scheme. The Spearman rank correlation of the theorem-aligned composite against the baseline ranking is ≥ 0.999 across all schemes—exactly $\rho = 1.000$ under the mean-preserving $K = 10$ and $K = 20$ refinements (consistent with the analytic invariance of an expectation under mean-

preserving refinement), with the small departures coming only from the severity-anchor perturbations; the entropy-enriched composite is slightly more sensitive (≥ 0.988). Full results are in `revision/outputs/R7_granularity.csv`.

6. S5. Condition Registry

6.1. eTable S1. Complete Condition List with Diagnostic Urgency Scores

The following table lists all 42 conditions in the diagnostic urgency registry, together with the primary theorem-aligned composite and the component scores used to compute it. Five conditions (marked with †) were excluded from the MIMIC-IV criterion-validity analysis due to insufficient inpatient representation in the critical care database. Dimension scores: Sev = expected missed-diagnosis severity, Entr = entropy-enriched trajectory comparator, I = irreversibility (expected permanent disability), R = directional preventable expected severity, S = time-sensitivity (hyperbolic decay of treatment half-life). Composite = arithmetic mean of Sev, I, R, and S. Entr is shown for comparison with the exploratory entropy-enriched score. All scores are normalized to [0, 1].

Parameter values for T, I, R, S were derived from published clinical literature (GBD 2019, Cochrane reviews, published case series) and are independent of the MIMIC-IV outcome data used for validation.

Conditions are ordered by composite urgency score (highest to lowest).

Rank	Condition	Cluster	Composite	Sev	Entr	I	R	S
1	Tension Pneumothorax	chest	0.721	1.00	0.30	0.00	1.00	0.88
2	Ruptured AAA	abdominal	0.528	0.92	0.50	0.13	0.51	0.55
3	Cerebellar Hemorrhage	dizziness	0.525	0.64	0.99	1.00	0.24	0.22
4	Mesenteric Ischemia	abdominal	0.464	0.82	0.77	0.35	0.46	0.22
5	Aortic Dissection	chest	0.399	0.76	0.85	0.19	0.28	0.37
6	Cardiac Arrhythmia (VT/VF)	syncope	0.387	0.37	0.84	0.08	0.09	1.00
7	Epidural Abscess	fever	0.385	0.40	0.89	0.85	0.17	0.12
8	Posterior Circulation Stroke	dizziness	0.368	0.45	0.96	0.69	0.06	0.28
9	Subarachnoid Hemorrhage	headache	0.356	0.66	0.96	0.46	0.22	0.08
10	Anaphylaxis	dyspnea	0.320	0.21	0.63	0.00	0.19	0.88
11	STEMI	chest	0.318	0.52	0.99	0.26	0.05	0.44
12	Necrotizing Fasciitis	fever	0.290	0.59	1.00	0.19	0.22	0.16
13	Bacterial Meningitis	headache	0.282	0.47	0.94	0.29	0.15	0.22
14	Esophageal Rupture (Boerhaave)	chest	0.269	0.61	0.98	0.08	0.22	0.16
15	Sepsis	fever	0.230	0.47	0.94	0.20	0.06	0.18
16	Aortic Stenosis (Critical)	syncope	0.196	0.39	0.91	0.26	0.13	0.01
17	Endocarditis	fever	0.191	0.44	0.94	0.19	0.11	0.02
18	Temporal (Giant Cell) Arteritis	headache	0.180	0.20	0.50	0.43	0.04	0.04
19	Vertebral Artery Dissection	dizziness	0.179	0.32	0.80	0.34	0.03	0.02
20	Pulmonary Embolism	chest	0.169	0.44	0.89	0.04	0.11	0.08
21	Acute CHF Exacerbation	dyspnea	0.167	0.25	0.70	0.32	0.01	0.08
22	COPD Exacerbation	dyspnea	0.127	0.21	0.61	0.24	0.01	0.04
23	Asthma Exacerbation	dyspnea	0.127	0.13	0.44	0.01	0.00	0.37
24	Pneumonia (Severe)	dyspnea	0.115	0.29	0.78	0.02	0.03	0.12
25	Ovarian Torsion	abdominal	0.114	0.19	0.49	0.06	0.08	0.12
26	Ectopic Pregnancy	abdominal	0.110	0.18	0.58	0.02	0.08	0.16
27	Bowel Obstruction	abdominal	0.100	0.26	0.73	0.06	0.04	0.04
28	NSTEMI/Unstable Angina	chest	0.094	0.27	0.72	0.07	0.00	0.04
29	Cerebral Venous Sinus Thrombosis	headache	0.089	0.26	0.75	0.04	0.03	0.02
30	Myocarditis	chest	0.060	0.19	0.59	0.04	0.01	0.00
31	Pancreatitis	abdominal	0.050	0.16	0.52	0.04	0.00	0.00
32	Appendicitis	abdominal	0.047	0.14	0.42	0.00	0.02	0.03
33	Pericarditis	chest	0.038	0.12	0.38	0.01	0.02	0.00
34	Cholecystitis	abdominal	0.036	0.12	0.38	0.00	0.00	0.02
35	Pyelonephritis	fever	0.033	0.07	0.25	0.01	0.03	0.02

Rank	Condition	Cluster	Composite	Sev	Entr	I	R	S
36	Migraine	headache	0.013	0.05	0.10	0.00	0.01	0.00
37	Vestibular Neuritis †	dizziness	0.011	0.03	0.06	0.01	0.00	0.00
38	GERD	chest	0.010	0.02	0.05	0.01	0.02	0.00
39	BPPV †	dizziness	0.008	0.01	0.01	0.00	0.02	0.00
40	UTI (Uncomplicated) †	fever	0.007	0.01	0.03	0.00	0.02	0.00
41	Tension Headache †	headache	0.004	0.01	0.01	0.00	0.01	0.00
42	Musculoskeletal Chest Pain †	chest	0.003	0.00	0.00	0.00	0.01	0.00

† Excluded from MIMIC-IV validation due to insufficient inpatient representation.

6.2. eTable S1b. Parameter Provenance Schema

The full provenance file is `research/outputs/tables/tableS1_data_sources.csv`. Each condition-component estimate is traceable to a literature or dataset source through the following fields.

Field	Meaning
condition	Diagnostic condition
component	Severity, entropy comparator, irreversibility, treatability, or time-sensitivity
point_estimate	Value used in the primary score before normalization
scale_direction	Whether larger values indicate greater urgency
source_type	GBD estimate, systematic review, randomized trial, registry, case series, guideline, or expert-imputed proxy
source_citation_or_dataset	Citation or dataset supporting the estimate
extraction_rule	Rule used to convert the source into a score
uncertainty_low	Lower plausible value when available
uncertainty_high	Upper plausible value when available
confidence_grade	High, moderate, low, or expert-derived
notes	Free-text qualification

Where source-level uncertainty is unavailable, sensitivity analyses vary component weights and leave-one-component-out structure rather than treating the point estimate as known without error.

6.3. eTable S2. Chief Complaint Cluster Assignments

Conditions are grouped by chief complaint cluster, reflecting the clinical presentation pathway through which patients enter the emergency department.

Cluster	Conditions
**chest_pain** (10)	STEMI, NSTEMI, Pulmonary Embolism, Aortic Dissection, Tension Pneumothorax, Pericarditis, Myocarditis, Esophageal Rupture, GERD, MSK Chest Pain
**dizziness** (5)	Posterior Stroke, BPPV, Vestibular Neuritis, Cerebellar Hemorrhage, Vertebral Dissection
**headache** (6)	SAH, Bacterial Meningitis, Temporal Arteritis, CVST, Migraine, Tension Headache
**abdominal** (8)	Ruptured AAA, Appendicitis, Bowel Obstruction, Mesenteric Ischemia, Pancreatitis, Cholecystitis, Ectopic Pregnancy, Ovarian Torsion
**dyspnea** (5)	Anaphylaxis, Severe Pneumonia, Asthma Exacerbation, Acute CHF, COPD Exacerbation
**syncope** (2)	Cardiac Arrhythmia VT/VF, Aortic Stenosis
**fever** (6)	Sepsis, Endocarditis, Necrotizing Fasciitis, Epidural Abscess, Pyelonephritis, UTI

The cluster structure serves two purposes: (1) it groups conditions that share a common initial presentation, reflecting the clinical reality that the emergency physician begins with a chief complaint and must differentiate among the conditions within that cluster; and (2) it enables within-cluster validation, testing whether the urgency ranking correctly orders the differential diagnosis for each presenting complaint.

6.4. eTable S3. Acuity Reference Labels for the Discrimination Analysis

The discrimination analysis (main text Section 6.2.1) tests whether the theorem-aligned urgency score separates emergent from lower-acuity conditions. Each of the 42 conditions was assigned an acuity reference label by author clinical consensus, **independently of the urgency score and of MIMIC-IV outcomes**, using the operational criterion: *emergent* = an immediate threat to life, limb, or major organ if confirmation or exclusion is delayed beyond hours (Emergency Severity Index level 1–2 equivalent); *lower-acuity* = not immediately life-threatening on this time scale. The full labeling is listed below for transparency and reviewer scrutiny (28 emergent, 14 lower-acuity). The theorem-aligned score discriminates the two classes with AUC = 0.96 (entropy-enriched comparator AUC = 0.98); the result is robust to single boundary-label flips (AUC range 0.95–0.98). The five conditions marked "Yes" under *MIMIC-excluded* are the low-acuity conditions omitted from the MIMIC outcome analysis; all five fall in the bottom 14% of the score distribution.

Condition	Composite	Acuity label	MIMIC-excluded
Tension Pneumothorax	0.721	Emergent	
Ruptured AAA	0.528	Emergent	
Cerebellar Hemorrhage	0.525	Emergent	
Mesenteric Ischemia	0.464	Emergent	
Aortic Dissection	0.399	Emergent	
Cardiac Arrhythmia (VT/VF)	0.387	Emergent	
Epidural Abscess	0.385	Emergent	
Posterior Circulation Stroke	0.368	Emergent	
Subarachnoid Hemorrhage	0.356	Emergent	
Anaphylaxis	0.321	Emergent	
STEMI	0.318	Emergent	
Necrotizing Fasciitis	0.290	Emergent	
Bacterial Meningitis	0.282	Emergent	
Esophageal Rupture (Boerhaave)	0.269	Emergent	
Sepsis	0.230	Emergent	
Aortic Stenosis (Critical)	0.196	Emergent	
Endocarditis	0.191	Emergent	
Temporal (Giant Cell) Arteritis	0.180	Emergent	
Vertebral Artery Dissection	0.179	Emergent	
Pulmonary Embolism	0.169	Emergent	
Acute CHF Exacerbation	0.167	Emergent	
COPD Exacerbation	0.127	Lower-acuity	
Asthma Exacerbation	0.127	Lower-acuity	
Pneumonia (Severe)	0.115	Emergent	
Ovarian Torsion	0.114	Emergent	
Ectopic Pregnancy	0.110	Emergent	
Bowel Obstruction	0.101	Emergent	
NSTEMI/Unstable Angina	0.094	Emergent	
Cerebral Venous Sinus Thrombosis	0.089	Emergent	
Myocarditis	0.060	Emergent	
Pancreatitis	0.050	Lower-acuity	
Appendicitis	0.047	Lower-acuity	
Pericarditis	0.038	Lower-acuity	
Cholecystitis	0.036	Lower-acuity	
Pyelonephritis	0.033	Lower-acuity	
Migraine	0.013	Lower-acuity	
Vestibular Neuritis	0.011	Lower-acuity	Yes
GERD	0.011	Lower-acuity	
BPPV	0.008	Lower-acuity	Yes
UTI (Uncomplicated)	0.007	Lower-acuity	Yes
Tension Headache	0.004	Lower-acuity	Yes
Musculoskeletal Chest Pain	0.003	Lower-acuity	Yes

Boundary conditions (those whose label a reviewer might reasonably contest: appendicitis, cholecystitis, pancreatitis, pericarditis, COPD/asthma exacerbation, myocarditis, severe pneumonia) were the targets of the flip-robustness check; reclassifying any single one leaves AUC ≥ 0.95.

6.5. References

Debreu G. Topological methods in cardinal utility theory. In: Arrow KJ, Karlin S, Suppes P, eds. *Mathematical Methods in the Social Sciences*. Stanford University Press; 1960:16-26.

GBD 2019 Diseases and Injuries Collaborators. Global burden of 369 diseases and injuries in 204 countries and territories, 1990-2019. *Lancet*. 2020;396(10258):1204-1222.

Keeney RL, Raiffa H. *Decisions with Multiple Objectives: Preferences and Value Tradeoffs*. Wiley; 1976.

Krantz DH, Luce RD, Suppes P, Tversky A. *Foundations of Measurement*. Vol 1. Academic Press; 1971.

Munkres JR. *Topology*. 2nd ed. Prentice Hall; 2000.

Wakker PP. *Additive Representations of Preferences: A New Foundation of Decision Analysis*. Kluwer; 1989.

Disclaimer/Publisher's Note: The statements, opinions and data contained in all publications are solely those of the individual author(s) and contributor(s) and not of MDPI and/or the editor(s). MDPI and/or the editor(s) disclaim responsibility for any injury to people or property resulting from any ideas, methods, instructions or products referred to in the content.
